# DIS3 licenses B cells for plasma cell differentiation in humans

**DOI:** 10.1038/s41423-025-01369-5

**Published:** 2025-11-25

**Authors:** Emma Miglierina, Julien Bouder, Delfina Ordanoska, Maïwenn Pineau, Simon Léonard, Anaïs Schavgoulidze, Gwenaëlle Quéré, Maeva Le Goff, Maé Bouchet, Steve Alexandre Genebrier, Samuel Bastos Serra Trinca, Laurent Deleurme, Céline Monvoisin, Laure Derrier, Charles Dumontet, Laurent Delpy, Jérôme Moreaux, Jill Corre, Michel Cogné, Brice Laffleur

**Affiliations:** 1 Inserm, University of Rennes, EFS Bretagne, UMR 1236, CHU Rennes, Rennes, France; 2 IUCT Oncopole, Toulouse, France; 3 Inserm 1052/CNRS 5286, University of Lyon, Lyon, France; 4 University of Limoges, UMR CNRS 7276/Inserm 1262, Limoges, France; 5 Institute of Human Genetics, UMR CNRS 9002, Montpellier, France; 6 Present Address: University of Limoges, UMR CNRS 7276/Inserm 1262, Limoges, France

**Keywords:** DIS3, Plasma cell, Centromeric RNA (cenRNA), Class switch recombination, Genomic instability, Multiple myeloma, Humoral immunity, Cancer genomics

## Abstract

DIS3 is the main catalytic subunit of the nuclear RNA exosome, a complex playing a crucial role in RNA processing and the degradation of various noncoding RNA substrates. In mice, DIS3 is essential for genomic rearrangements during B cell development, but its role in terminal plasma cell (PC) differentiation has not been explored. Although *DIS3* gene alterations are frequent in multiple myeloma (MM), a PC malignancy, their molecular impact remains poorly understood. In this study, we developed an antisense oligonucleotide strategy to knock down *DIS3* expression in a well-characterized model of human PC differentiation. Reducing *DIS3* expression systematically led to decreased B cell proliferation and impaired PC differentiation with lower levels of switched immunoglobulin secretion. Transcriptome analyses confirmed alterations in the proliferation and differentiation programs, alongside an accumulation of noncoding RNAs. Notably, centromere-associated noncoding RNAs were highly sensitive to DIS3 activity, and their accumulation in DIS3-deficient cells, either as transcripts or DNA-associated RNAs, correlated with the mislocalization of the centromere-specific histone variant CENP-A. We finally observed reduced physiological DNA recombination and somatic hypermutation but increased genomic instability in DIS3-deficient cells, in agreement with the higher levels of *IGH* translocations observed in our large cohort of *DIS3*-mutant MM patients. Together, these results underscore the essential role of DIS3 in regulating B cell proliferation, DNA recombination, and physiological or malignant PC differentiation in humans.

## Introduction

DIS3 is a highly conserved ribonuclease and the main catalytic subunit of the nuclear RNA exosome, a machinery responsible for RNA processing, surveillance, and degradation [1]. This complex plays a crucial role in maintaining RNA homeostasis by targeting aberrant and noncoding (nc) RNAs for degradation [2]. The RNA exosome also contributes to the regulation of chromatin-associated RNAs [3], which can form potentially harmful R-loops, leading to DNA double-strand breaks (DSBs) and genomic instability [4].

In mice, DIS3 is essential for B cell development, and is particularly implicated in facilitating programmed DNA remodeling events, including V(D)J recombination [5]. Its role is especially critical during the germinal center (GC) reaction, where B cells undergo class switch recombination (CSR) [6] and somatic hypermutation (SHM) [7]. Beyond these genomic rearrangements that shape B cell receptors (BCRs), the impact of DIS3 on terminal PC differentiation is unknown.

PC differentiation is the ultimate step of B cell maturation and requires extensive rounds of cell division, at least 6 to 8 divisions [8]. PC differentiation dramatically reshapes not only the transcriptomic and epigenetic landscapes of these cells but also genome organization and cell morphology [9]. PCs are specialized in immunoglobulin (Ig) production, with an expanded secretory apparatus ensuring long-term humoral immunity. Extrafollicular PC differentiation mainly produces short-lived PCs, whereas the GC reaction gives rise to a subset of long-lived PCs [10]. In GCs, PC differentiation is initiated in the light zone by antigen-presenting dendritic cells and T follicular helper cells that trigger B cell activation followed by intense proliferation in the dark zone. Concomitant SHM ensures Ig affinity maturation for antigen and selected cells are finally licensed to differentiate into memory B cells (mBCs) or PCs [11]. In parallel, the mechanism of CSR changes Ig constant parts for optimal immune response. These genomic rearrangements expose the B cell genome to the mutagenic enzyme activation-induced cytidine deaminase (AID), which deaminates cytidines into uridines, which are then processed by the DNA repair machinery. In physiology, these events introduce on-target mutations in the V(D)J genes and DSBs at switch (S) regions of the IG heavy chain (*IGH*) locus followed by CSR [12]. In pathology, they generate off-target oncogenic mutations and DSBs that lead to translocations, frequently observed in B cell neoplasia and MM [13]. Overall, there is an intricate relationship between B cell proliferation, SHM, CSR, and PC differentiation.

In humans, four *DIS3* transcript isoforms coexist in a tight equilibrium that is altered in hematological disorders, including MM [14]. Additionally, the *DIS3* gene is often damaged in MM; one copy is frequently lost (~45% of the patients) with deletions affecting the chromosome 13 [15–17], and somatic mutations are observed in 10-15% of patients [17–19], usually disrupting DIS3 enzymatic activity [20]. Furthermore, germline variants affecting DIS3 catalytic properties predispose patients to develop familial MM [21]. Patients with double hits of the *DIS3* gene (a deletion associated with a mutation or two mutations) have the worst prognosis [18, 22], while double deletions have not been identified, likely because this gene is essential for cell viability [23]. DIS3 loss-of-function (LOF) is predominantly associated with PC diseases, including monoclonal gammopathy of undetermined significance (MGUS), smoldering MM (SMM), MM, and PC leukemia [17, 24, 25]. MM is an incurable cancer of PCs characterized by abnormal PC accumulation in the bone marrow and a high level of genomic instability [26].

DIS3 expression and enzymatic activities are thus necessary for physiology but are altered in pathology, although the underlying mechanisms remain insufficiently understood.

In this study, we developed an antisense oligonucleotide (ASO) strategy to knock down DIS3 expression in a well-characterized model of in vitro PC differentiation, using human primary B cells from healthy donors [27–29]. This approach revealed the critical role of DIS3 for cell proliferation, physiological PC differentiation, and associated secretion of switched Igs. In the absence of DIS3, the coding and noncoding transcriptomes were altered, and we identified a substantial accumulation of centromere-derived ncRNAs (cenRNAs) coinciding with mislocalization of CENP-A, a key centromere-specific histone variant, that may contribute to decreased proliferation. R-loop accumulation was detected at centromeres, likely contributing to this phenotype, and potentially weakening genome integrity of DIS3-deficient cells. In agreement, we observed lower physiological DNA recombination but higher genomic instability in DIS3-deficient cells. Together, these data demonstrate an essential role for DIS3 in ensuring physiological PC differentiation while avoiding the pathological alterations observed in MM patients with *DIS3* mutations.

## Results

### *DIS3* expression in pathophysiological plasma cells

**Fig. 1:**
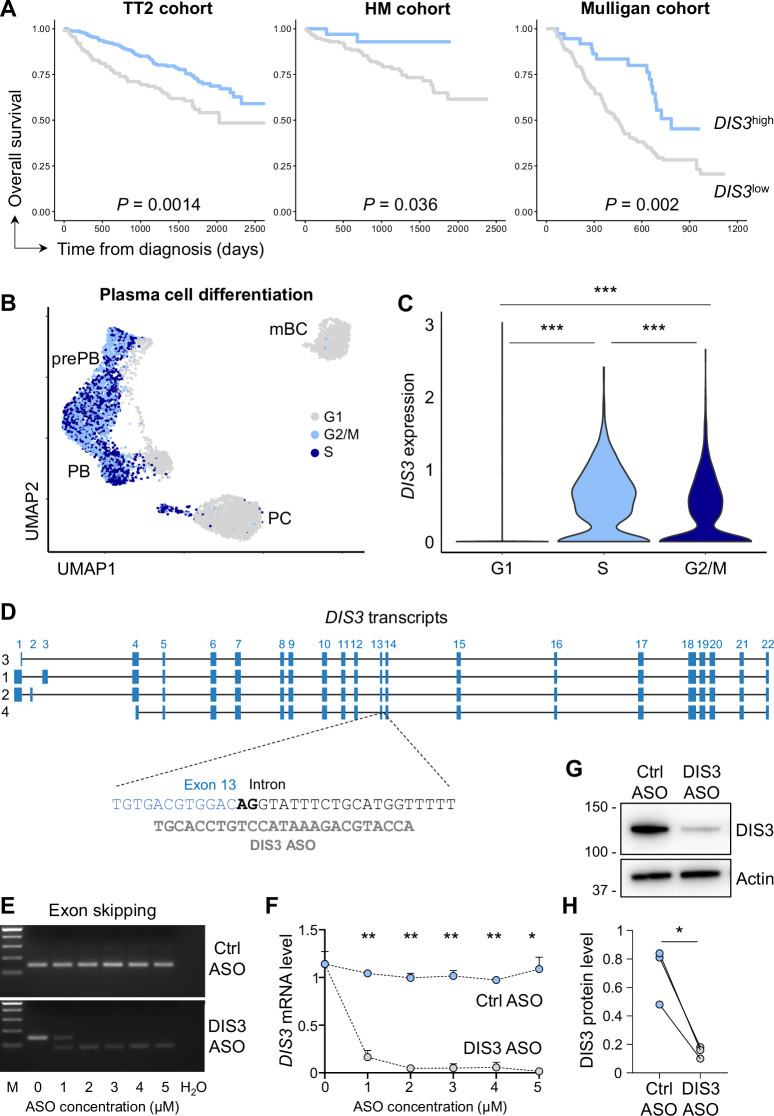
*DIS3* expression in pathophysiological plasma cells. **A** *DIS3* gene expression and associated overall survival in MM patients. Data from 3 independent cohorts (TT2: *n* = 345: 111 *DIS3*^low^, 234 *DIS3*^high^; HM: *n* = 206: 173 *DIS3*^low^, 33 *DIS3*^high^; Mulligan: *n* = 188: 151 *DIS3*^low^, 37 *DIS3*^high^). The association between *DIS3* gene expression and overall survival was analyzed using the Maxstat R package, *P*-values are shown. **B** Uniform manifold approximation and projection (UMAP) of single-cell RNA sequencing displaying cell cycle-associated clusters during in vitro PC differentiation. **C** *DIS3* expression in cell cycle-associated clusters during in vitro PC differentiation. Wilcoxon statistical tests, *n* = 3589 cells in G1, *n* = 1481 cells in S, and *n* = 1322 cells in G2/M phase. **D** *DIS3* transcript isoforms and DIS3 ASO, targeting the exon 13 splice donor site (in bold), are represented. **E** SSK41 B cells were treated with ctrl or DIS3 ASOs for 72 h. RT-PCRs were performed using oligos located in exons 12 and 14. Electrophoresis gels show accurate splicing between exons 12, 13, and 14 (174 bp) in controls while DIS3 ASOs induced exon skipping and splicing from exon 12 to 14 (81 bp). Data are representative from 3 independent experiments, *n* = 3. **F** Splicing between exons 13 and 14 was evaluated by RT-qPCR. Means ± s.e.m. are shown, two-tailed paired *t*-test, 3 independent experiments, *n* = 3. **G** Western blot analyses of DIS3 protein expression after 3 µM ASO treatments in SSK41 B cells. Data are representative of 3 independent experiments, *n* = 3. **H** Quantification from Western blots of DIS3 protein expression after ASO treatments in SSK41 B cells. Individual values are shown, two-tailed paired *t*-test, 3 independent experiments, *n* = 3

Alterations of the *DIS3* gene have been described in MM, however, the expression level of *DIS3* transcripts in patients has been less explored. We evaluated *DIS3* expression in PCs of MM patients from multiple cohorts, including two cohorts of newly diagnosed MM patients treated with high doses of melphalan and autologous stem cell transplantation (total therapy 2 (TT2) and Heidelberg-Montpellier (HM)), and a cohort of patients at relapse treated with bortezomib monotherapy (Mulligan cohort). In each case, we observed a statistically significant better prognosis for patients with high compared to those with low *DIS3* expression (Fig. 1A). Accordingly, when ranking patients according to disease evolution, significantly lower *DIS3* expression was observed in the groups of patients with bad prognoses (Fig. S1A). These data suggest a contribution of DIS3 to MM severity, where low *DIS3* expression may result from allelic loss and/or invalidating mutations of the *DIS3* gene, or epigenetic alterations. We then investigated *DIS3* expression during physiological PC differentiation in humans. We analyzed single-cell RNA-sequencing data from the in vitro PC differentiation model [29] and found detectable *DIS3* expression mainly in clusters 2, 3, and 4, corresponding to preplasmablasts (prePBs) and plasmablasts (PBs) (Fig. S1B–D). Furthermore, *DIS3* expression was predominantly observed in actively cycling cells, during the S and G2/M phases (Fig. 1B, C), suggesting that DIS3 plays a role in facilitating the cell cycle progression necessary for PC differentiation. In parallel, we analyzed single-cell RNA-sequencing data from sorted in vivo bone marrow PCs [30], and similarly, we observed higher *DIS3* expression in PC progenitors that highly expressed the proliferation marker *MKI67* (Fig. S1E–H).

To directly investigate the role of DIS3 in the PC differentiation process, we developed a strategy to knock down *DIS3* expression in the human B cell lineage. We followed an efficient ASO-based approach to target pathophysiological PCs [28, 31] and designed a DIS3 ASO that targets all *DIS3* isoforms (Fig. 1D). The DIS3 ASO hybridizes to the exon 13-intron junction, masking the donor splice site, inducing exon skipping, and generating unstable out-of-frame *DIS3* transcripts. We treated the B cell line SSK41 with increasing concentrations of ASOs and confirmed exon skipping by RT-PCR. In control cells, we detected correct splicing of exons 12-13-14 after control (ctrl) ASO treatments, whereas DIS3 ASOs redirected splicing from exon 12 to 14, as expected, with ASO concentrations as low as 2 µM (Fig. 1E). We quantified functional *DIS3* transcripts (exons 13–14 splicing junction) by RT-qPCR and observed an approximate decrease of 95% with the 2 µM dose under these conditions (Fig. 1F). Finally, we evaluated the DIS3 protein level by Western blot and detected ~80% decrease with a 3 µM of DIS3 ASO treatment compared to the controls in SSK41 cells (Fig. 1G, H), and a significant decrease in primary B cells (Fig. S1I, J).

Therefore, DIS3 ASOs represent a suitable tool to knock down *DIS3* expression in the human B cell lineage, mimicking low *DIS3* expression observed in a subset of MM patients, and offering the opportunity to study the role of DIS3 during PC differentiation.

### DIS3 is essential for B cell proliferation and plasmablast differentiation

**Fig. 2:**
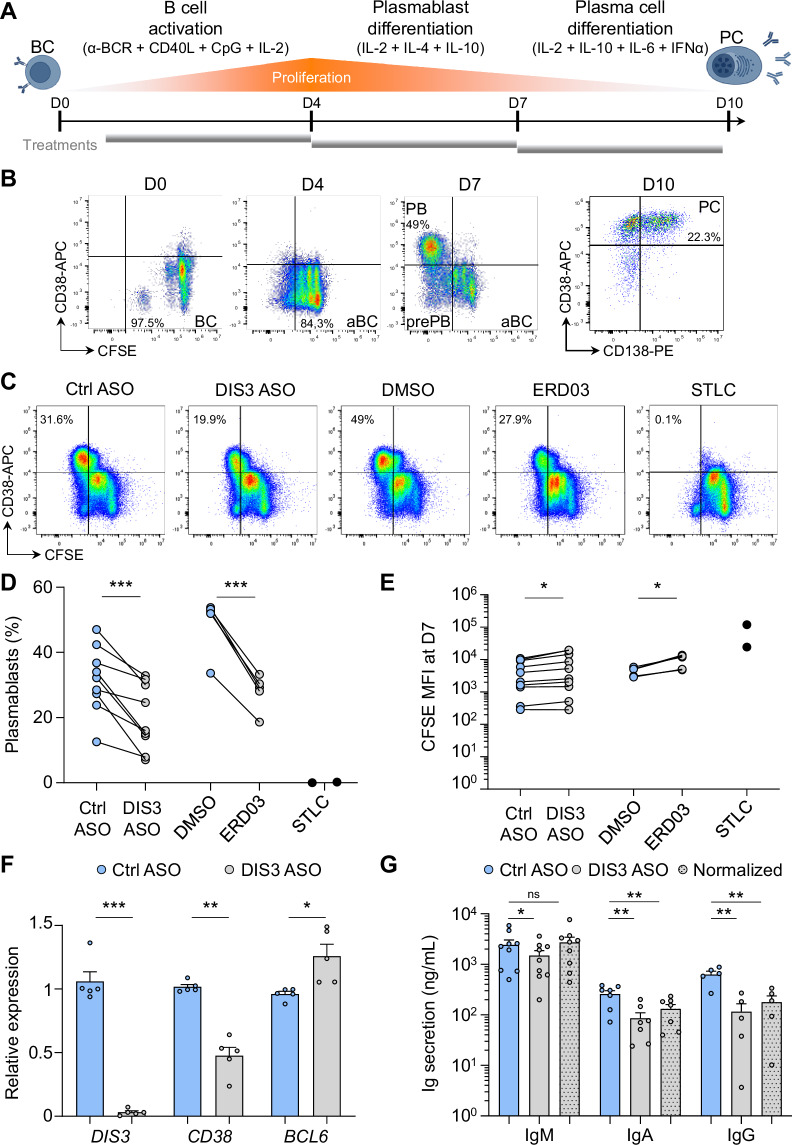
DIS3 is essential for B cell proliferation and plasmablast differentiation. **A** PC differentiation protocol. B cells were sorted from buffy coats and labeled with CFSE at D0. A first step of activation was performed between D0 and D4, followed by a second step of differentiation between D4 and D7, allowing cell proliferation and PB differentiation. A last step between D7 and D10 induced PC differentiation. ASO and other treatments were performed independently during these different steps for 3 days. **B** Monitoring of PC differentiation by flow cytometry. CFSE dilution and CD38 expression were quantified between D0 and D7, followed by CD38 and CD138 expression at D10. **C** DIS3 inhibition decreases PB differentiation. Activated B cells were treated from D4 to D7 with ctrl or DIS3 ASOs, DMSO or ERD03, or STLC and analyzed by flow cytometry. **D** Quantification of PB percentage. Individual values are shown, two-tailed paired *t*-test, at least 5 independent experiments, *n* = 2– 9. **E** CFSE MFIs at D7. Individual values are shown, two-tailed paired *t*-test, at least 5 independent experiments, *n* = 2–9. **F** RT-qPCR analyses at D7. Means ± s.e.m. and individual values are shown, two-tailed paired *t*-test, 5 independent experiments, *n* = 5. **G** IgM, IgA, and IgG secretion were evaluated by ELISA at D7. Samples treated with DIS3 ASOs were also normalized to the same cell number as the ctrl ASOs (dotted gray bars). Means ± s.e.m. and individual values are shown, two-tailed paired *t*-test, at least 5 independent experiments, *n* = 5–8

Human PC differentiation can be achieved efficiently in vitro [27–29]. In this system, B cells are purified from buffy coats of healthy donors and stimulated for 4 days with a cocktail of activators (anti-BCR, CD40 ligand (CD40L), CpG oligodeoxynucleotides, and interleukin (IL)-2). During this step, resting B cells are first activated and initiate proliferation. The media is changed at day (D) 4 and cells are treated with cytokines (IL-2, IL-4, and IL-10) for 3 days, inducing multiple cycles of cell division followed by PB differentiation. Finally, the media is replaced by new cytokines (IL-2, IL-6, IL-10, and interferon (IFN)-α) from D7 to D10 to enable the final step of PC differentiation, which is less proliferation-dependent. This protocol has been extensively validated, and the time points have been determined to ensure optimal B cell activation followed by PB and PC differentiation [27–29]. We further optimized this model by sorting total B cells from buffy coats and labeled them at D0 with CFSE to monitor proliferation. We then performed ASO treatments for 3 days, either from D1 to D4, from D4 to D7, or from D7 to D10, to allow sufficient time for mRNA knockdown and subsequent protein depletion (Fig. 2A). PC differentiation was monitored by flow cytometry, following CFSE dilution and expression of CD38 for PB differentiation and CD138 for PC differentiation (Fig. 2B). In these experiments, we included various treatments: S-trityl-L-cysteine (STLC), a cell cycle inhibitor; ERD03, a chemical inhibitor of the nuclear and cytoplasmic RNA exosomes [32]; and QVD-OPH, a pan-caspase inhibitor.

During the early step of B cell activation (until D4), DIS3 ASOs consistently delayed CFSE dilution, i.e. cell division (Fig. S2A-B). Accordingly, CFSE mean fluorescent intensity (MFI) was increased in the absence of DIS3 (Fig. S2C), while cell number was globally unaffected (Fig. S2D). As a control, STLC strongly impacted CFSE dilution, as expected (Fig. S2A–D). We also totally abrogated RNA exosome activity with ERD03 and noticed lower B cell proliferation, mirroring the phenotype observed with DIS3 ASOs (Fig. S2A-D).

Strikingly, between D4 and D7, we systematically observed a substantial decrease in proliferation following DIS3 ASO treatments, resulting in an accumulation of CFSE^high^ CD38^-^ activated B cells (aBCs) while the frequency of CFSE^low^ CD38^+^ PBs was decreased (Fig. 2C–E). DIS3 inhibition in the presence of QVD-OPH induced a similar trend with fewer PBs (Fig, S2E), suggesting that the differentiation defect did not result from intense apoptosis of the cells. Chemical inhibition of the RNA exosome with ERD03 also consistently perturbed proliferation and decreased PB differentiation compared to the DMSO control (Fig. 2C-E). Furthermore, the absolute number of PBs was strongly decreased in the absence of RNA exosome activity, while the number of aBCs was unaffected (Fig, S2F, G). As controls, STLC treatments induced a massive accumulation of aBCs with virtually no detectable PBs (Fig. 2C–E), confirming that PB differentiation is strictly dependent on proliferation. We then collected cells for RNA extraction and culture supernatants for evaluating Ig secretion at D7. Transcriptomic analyses confirmed efficient *DIS3* inhibition and reflected the differentiation defect, with lower expression of the PB-associated transcript *CD38*, while the aBC-associated *BCL6* transcript accumulated (Fig. 2F). ELISA analyses revealed lower Ig secretion, with a ~ 40% decrease in IgM, a ~ 66% decrease in IgA, and a ~ 75% decrease in IgG production (Fig. 2G). We normalized this secretion to the same cell number as the control and still observed a significant decrease in IgA and IgG secretion (Fig. 2G). This altered secretion of switched Ig may result from the PB differentiation defect, CSR deficiency, and/or intrinsic alteration of protein secretion in the absence of DIS3.

Finally, we treated cells during the last step of PC differentiation, between D7 and D10. At this stage, which is less dependent on cell proliferation, DIS3 ASOs and ERD03 treatments only slightly affected PC generation, and impacted rather the final number of PCs than the PC differentiation efficiency itself (Fig. S2H–K). As a consequence, lower levels of Igs were quantified by ELISA, with about a 50% decrease for IgM, IgA, and IgG secretion, but this effect was abolished after normalization to the same cell number as the control (Fig. S2L).

In parallel, we analyzed data from two independent screening studies that identified all the subunits of the RNA exosome [33] or *Dis3* [34] to be essential for B cell survival/proliferation and PC differentiation in mice (Fig. S2M, N). We directly confirmed these data by targeting mouse B cells with Dis3 ASOs, and again observed fewer B220^low^ CD138^+^ PBs in the absence of DIS3 (Fig. S2O, P).

In view of these elements, we concluded that DIS3 is critical for PC differentiation, especially for the early steps of intense B cell proliferation and associated PB differentiation.

### DIS3 alterations reshape transcriptomic landscapes

To decipher DIS3-associated molecular mechanisms involved in PC differentiation, we sequenced RNAs from bulk cells and sorted subpopulations. Bulk cells were collected on day 6 (D6), 48 h after ASO treatments; and CFSE^high^ CD38^-^ aBCs, CFSE^low^ CD38^-^ prePBs, and CFSE^low^ CD38^+^ PBs were sorted on day 7, 72 h after ASO treatments. Deep strand-specific RNA-sequencing was performed on total RNAs after ribodepletion on ctrl and DIS3 ASO-treated cells from 4 donors at D6 and another 3 independent donors at D7.

**Fig. 3:**
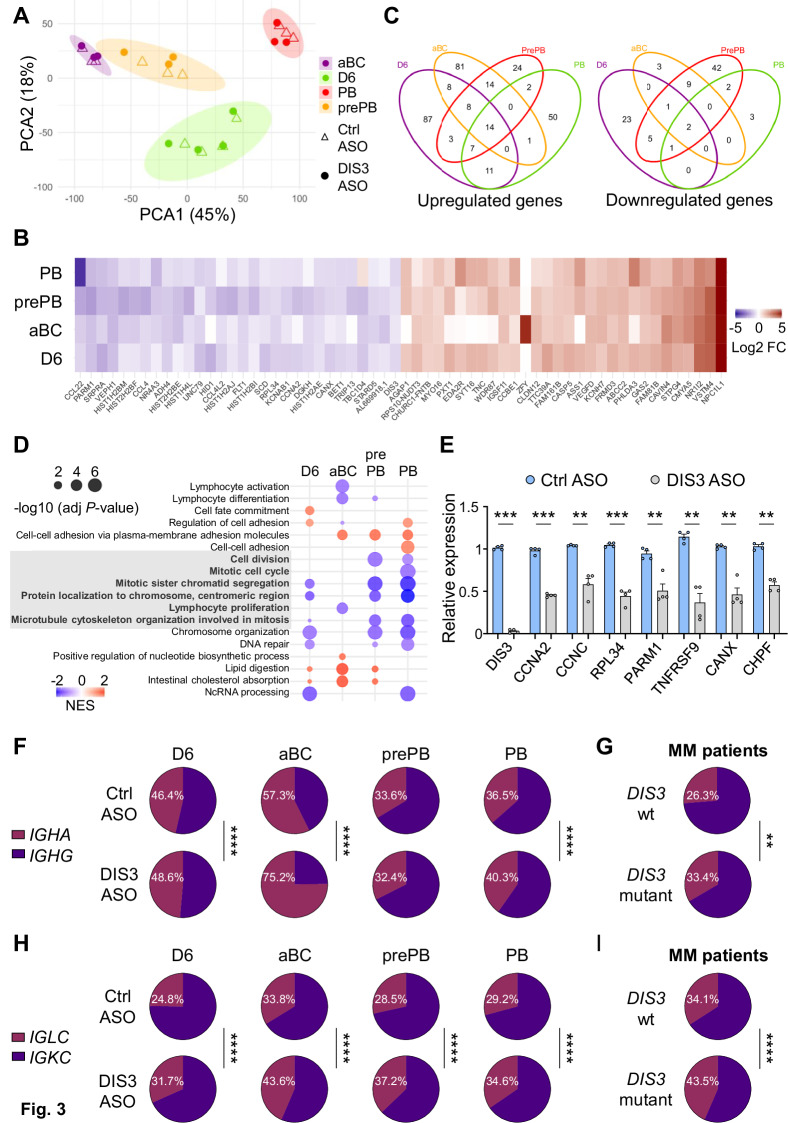
DIS3 alterations reshape transcriptomic landscapes. **A** PCA analyses from differentiating cells, from bulk D6 cells (*n* = 4) or D7 sorted aBCs (*n* = 3), prePBs (*n* = 3), and PBs (*n* = 3). **B** Heatmaps showing fold changes (FC) of differentially expressed genes at D6, or at D7 in aBCs, prePBs, and PBs, treated with ctrl versus DIS3 ASOs. The 30 most downregulated and most upregulated genes are shown across conditions, based on the average log2FC with adjusted *P*-values < 0.05. **C** Venn diagrams displaying differentially expressed genes (|log2FC| > 1), adjusted *P*-values < 0.05) in the different populations and their overlaps. **D** Gene set enrichment analyses (GSEA) of biological processes in the different populations. The -log10 of adjusted *P*-values (FDR correction) and the normalized enrichment score (NES) are represented. A negative NES (blue dots) indicates enrichment in downregulated genes, while a positive NES (red dots) indicates enrichment in upregulated genes. Pathways involved in proliferation are highlighted in gray. **E** Validation by RT-qPCR of differentially expressed genes in differentiating cells at D6. Individual values and means ± s.e.m. are shown, two-tailed paired *t*-test, 4 independent experiments, *n* = 4. **F** Repertoire analyses of *IGHA* and *IGHG* transcripts usage in the indicated populations. Two-sided Khi^2^ proportion test. **G** Data from MM patients showing *IGHA* and *IGHG* transcripts usage in control (*DIS3* wt, *n* = 2452) versus *DIS3* mutant patients (*n* = 392). Two-sided Khi^2^ proportion test. **H** Repertoire analyses of *IGL* transcripts usage in the indicated populations. Two-sided Khi^2^ proportion test. **I** Data from MM patients showing *IGL* transcripts usage in control (*DIS3* wt, *n* = 3056) versus *DIS3* mutant patients (*n* = 515). Two-sided Khi^2^ proportion test

We first investigated the impact of DIS3 knockdown on the coding transcriptome. Principal component analysis (PCA) showed the distinct signature of each population, as expected, while DIS3 knockdown did not drastically alter this distribution based on gene expression (Fig. 3A). Differential gene expression analysis for each condition revealed that only a limited number of coding transcripts were altered by DIS3 deficiency. 138, 126, 72, and 85 transcripts were upregulated, and 32, 15, 62, and 8 transcripts were downregulated after DIS3 ASO treatments in D6 cells, aBCs, prePBs, and PBs respectively (fold changes >2 or <0.5, adjusted *P*-values < 0.05, Figs. 3B, C, S3A, B, and Table 1). While some genes were differentially expressed in all populations, others were condition-specific (Fig. 3B, C).

Gene set enrichment analyses (GSEA) confirmed the alteration of biological pathways related to cellular activation and differentiation for the D6, aBCs, and prePBs precursor populations (Fig. 3D, Table 2). Importantly, cell cycle and proliferation pathways were largely impacted in all populations, in line with the involvement of DIS3 in cell proliferation (Fig. 3D, highlighted in grey). The underlying mechanisms may involve the microtubule cytoskeleton and protein localization to the centromeres, two mechanisms critical for mitosis that were altered by DIS3 deficiency (Fig. 3D). Other biological functions were affected, including DNA organization and repair, lipid metabolism, as well as RNA processing, as expected in the context of DIS3 ribonuclease deficiency (Fig. 3D). We evaluated B cell and PC master gene expression (*BACH2*, *BCL6*, *PAX5*, *IRF4*, *PRDM1*, and *XBP1*) but did not observe significant change (Fig. S3C). While it is challenging to determine the individual contribution of each differentially expressed gene, variations in specific genes may contribute to impaired PB differentiation. Nevertheless, the disrupted biological pathways are consistent with the observed phenotype.

We then used the D6 RNAs for direct RT-qPCR validation of the downregulation of two members of the cyclin family, cyclin A2 (*CCNA2*), a protagonist of cell cycle progression, and cyclin C (*CCNC*). We also confirmed lower expression of ribosomal protein L34 (*RPL34*), implicated in cell proliferation and cancer [35]; prostate androgen-regulated mucin-like protein 1 (*PARM1*), a potential oncogene involved in proliferation and leukemia [36]; and chondroitin polymerizing factor (*CHPF*), another potential oncogene involved in cellular proliferation [37]. TNF receptor superfamily member 9 (*TNFRSF9*, alias *CD137, 4-1BB*), an inducible costimulatory receptor involved in lymphocyte metabolism and survival [38]; and calnexin (*CANX*), an endoplasmic reticulum chaperone critically involved in the development and function of PCs, notably by supporting the unfolded protein response (UPR) necessary for antibody secretion [39], were also decreased in DIS3-deficient cells (Fig. 3E).

We then reconstituted the V(D)J repertoires from the transcriptome of these cells using the TRUST4 algorithm [40]. We evaluated the frequencies of CDR3 out-of-frame junctions in each population and only observed a slight decrease in DIS3-deficient aBCs (Fig. S3D). The VDJ repertoire analyses did not reveal any bias toward a particular segment for the *IGH* variable (IGHV) or junction (IGHJ) genes (Fig. S3E, F). The same was true for the VJ repertoires of the light chains, with no alteration of the V and J gene usage for the κ (IGKV and IGKJ) or λ (IGLV and IGLJ) locus (Fig. S3G–J).

By contrast, *IGH* constant genes were affected by DIS3 deficiency, where the abundance of *IGHM* transcripts was increased (Fig. S3K), likely reflecting defective CSR occurring during this phase of activation [27]. We then focused on switched transcripts and quantified the proportions of *IGHA* and *IGHG* transcripts, enabling the production of IgA and IgG antibodies. Unexpectedly, we observed an increased proportion of *IGHA* transcripts in DIS3-deficient cells, especially in aBCs (Fig. 3F).

In parallel, we studied a large cohort of newly diagnosed MM patients recruited within hospitals involved in the Intergroupe Francophone du Myélome, for whom next-generation sequencing (NGS) panel was performed as previously described [41] in the Unit of Genomics in Myeloma (Toulouse University hospital). We analyzed 3077 patients with unaltered *DIS3* gene (*DIS3* wild-type (wt)) and 520 patients with *DIS3* mutant gene, i.e. ~14% of *DIS3* mutations. For each patient, the following category of MM was available: light chain only, IGHA^+^, IGHG^+^, or other (including IGHM^+^, IGHD^+^, and IGHE^+^ rare myelomas). The proportion of patients with light chain myeloma did not differ in the group of MM patients, while the “other” category was slightly increased with *DIS3* mutations (Fig. S3L). Furthermore, the proportion of IgA MM cases was increased in the *DIS3* mutant group (Figs. 3G and S3L). This imbalance followed a common trend observed with primary cells (Fig. 3F) and may be due to preferential CSR to IgA in DIS3-deficient cells or cellular selection, potentially favoring the survival or expansion of IgA^+^ cells.

We then investigated *IG* light chain transcript expression in primary cells, and surprisingly, the *IGKC*/*IGLC* ratios were altered by DIS3 ASO treatments in each population (Fig. 3H). In parallel, we observed an increased usage of the lambda light chain in patients with *DIS3* mutations compared to the controls (Fig. 3I). This imbalance was therefore consistent in all evaluated conditions and suggests a selection bias of Igλ^+^ cells in the presence of *DIS3* alterations. We validated this observation by directly labelling primary cells at D7, demonstrating a significant increase of Igλ^+^ cells in ERD03-treated and DIS3-deficient cells from multiple donors (Fig. S3M, N).

Finally, we evaluated SHM levels in ctrl and DIS3 ASO-treated cells. To avoid any transcriptional bias, we collected genomic DNA at D7 to perform dedicated repertoire assays of the VDJ exons [42]. Bioinformatic analyses revealed reduced SHM rates in DIS3-deficient cells, affecting both silent and non-silent mutations (Fig. S3O–Q). We assessed repertoire diversity at CDR3 using the Hill numbers and observed no significant differences between groups for *q* = 0 to *q* = 3, reflecting comparable total richness, evenness, and clonal expansion patterns. However, a slight reduction in diversity at higher orders (*q* = 4 and *q* = 5) was detected in DIS3-deficient cells, indicating a modest increase in clonal dominance within the most abundant clones in this in vitro system (Fig. S3R).

To conclude, we observed variations in gene expression related to cell phenotype both in bulk cells and in individually sorted populations. Cell activation, proliferation, and differentiation were strongly impacted, as well as DNA repair, metabolism, and RNA processing, all of which may affect PC differentiation. IG repertoire analyses revealed alterations in the *IGHA*/*IGHG* transcript proportions, as well as *IGKC*/*IGLC*, reminiscent of MM patients with *DIS3* mutations, while DNA analyses demonstrated the critical role of DIS3 for optimal SHM in humans.

### Noncoding centromeric RNA accumulation in DIS3-deficient cells

The RNA exosome complex is mostly involved in the processing and decay of noncoding transcripts, suggesting that variations in coding gene expression might rather be a consequence of altered cellular physiology than a direct effect of DIS3 deficiency. By contrast, some ncRNAs are direct substrates and accumulate in the absence of RNA exosome activity [2]. Thus, we investigated ncRNA accumulation that may be related to the proliferation and differentiation defects. In that line, centromere-associated noncoding α-satellite RNAs (cenRNAs) are critical actors of cell cycle progression [43]. As gene ontology analyses pointed toward the biological pathway “protein localization to chromosome, centromeric region”, we hypothesized that these noncoding transcripts may be bona fide DIS3 substrates.

**Fig. 4:**
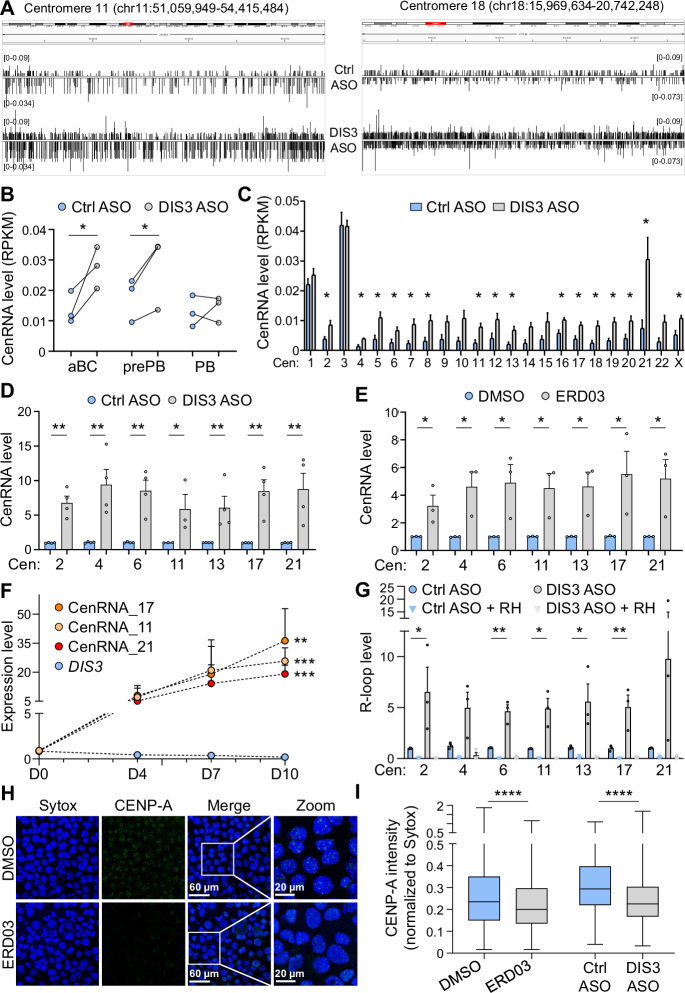
Noncoding centromeric RNA accumulation in DIS3-deficient cells. **A** IGV tracks from RNA-sequencing showing centromeric RNA accumulation at different chromosomes in aBCs treated with ctrl or DIS3 ASOs. Data are representative of 3 independent experiments, *n* = 3. **B** Quantification of cenRNA levels from RNA-sequencing of aBCs, prePBs, and PBs. Individual values are shown, one-tailed paired *t*-test, 3 independent experiments, *n* = 3. **C** Quantification of cenRNA levels from RNA sequencing data at individual centromere (cen) of aBCs. Means ± s.e.m. are shown, one-tailed paired *t*-test, 3 independent experiments, *n* = 3. **D** Quantification by RT-qPCR of cenRNA levels after ASO treatments at D7. Means ± s.e.m. and individual values are shown, two-tailed ratio paired *t*-test, 4 independent experiments, *n* = 3 to 4. **E** Quantification by RT-qPCR of cenRNA levels after ERD03 treatments at D7. Means ± s.e.m. and individual values are shown, two-tailed ratio paired *t*-test, 3 independent experiments, *n* = 3. **F** Quantification by RT-qPCR of cenRNA levels during in vitro PC differentiation in untreated cells. Means ± s.e.m. are shown, two-tailed ratio paired *t*-test, 5 independent experiments, *n* = 5. **G** Quantification by qPCR of centromeric R-loop levels after ASO treatments at D6. Means ± s.e.m. and individual values are shown, two-tailed ratio paired *t*-test, 3 independent experiments, *n* = 3. **H** Microscopy analyses of CENP-A levels in DMSO and ERD03 treated cells. SSK41 cells were stained intracellularly with anti-CENP-A antibodies, and DNA was stained with Sytox. Cells were analyzed by confocal microscopy. One image representative of 4 independent experiments, *n* = 4. **I** Quantification of CENP-A levels. CENP-A and Sytox signal intensities were quantified, and CENP-A intensity was normalized to Sytox. Box and whisker plots show the median, 25th to 75th percentiles and min to max values, two-tailed unpaired *t*-test, 4 independent experiments, 2968 cells and 3595 cells were analyzed for DMSO and ERD03 treatments in total, respectively; 3 independent experiments, 5524 cells and 3658 cells for ctrl and DIS3 ASO treatments, respectively

Using the visualization tool IGV, we examined ncRNA levels in the genome of aBCs and identified a massive accumulation of ncRNAs overlapping centromeres in DIS3-deficient cells, occurring both in sense and antisense orientations (Figs. 4A and S4A). To quantify cenRNA accumulation accurately, we mapped the sequencing reads to the telomere-to-telomere (T2T)-CHM13 genome [44], calculated centromeric coverage, and derived RPKM values. This analysis revealed an approximately 2-fold increase in cenRNA levels in aBCs and prePBs with DIS3 defect (Fig. 4B). This accumulation was consistent across most chromosomes, suggesting a general mechanism (Fig. 4C, and S4B, C). In parallel, we assessed other repetitive RNA transcripts, such as LINEs and LTRs, which did not significantly vary upon DIS3 depletion (Fig. S4D, E). We further examined the noncoding transcriptome of aBCs and observed a slight accumulation of other ncRNAs, including enhancer-associated RNAs (eRNAs), as expected in the absence of this ribonuclease. Importantly, we detected a significant eRNA accumulation at the super-enhancers of the *IGH* locus 3’ regulatory regions (3’RR1 and 3’RR2) (Fig. S4G, H), in agreement with the critical role of DIS3 at the *IGH* locus.

We next directly quantified cenRNAs expressed from various chromosomes by RT-qPCR. We used previously published assays [45] and also designed and validated new cenRNA primers overlapping RNA-sequencing signals. At D6, we confirmed the accumulation of cenRNAs at all studied chromosomes, with an approximate threefold increase in cells treated with DIS3 ASOs (Fig. S4F). This accumulation was even more substantial at D7, with at least a fivefold increase in DIS3-deficient primary B cells (Fig. 4D). We further confirmed this accumulation in cells treated with ERD03 (Fig. 4E). Overall, both RNA-sequencing and RT-qPCR experiments demonstrated a tremendous accumulation of cenRNAs in the absence of RNA exosome activity.

Independent of DIS3, we aimed to document more thoroughly the transcriptional regulation of these cenRNAs during PC differentiation. We thus collected RNA from untreated cells of our differentiation model at D0, D4, D7, and D10. We observed an important accumulation of cenRNAs, of about a 20-fold increase, in terminally differentiating cells stimulated by cytokines (D10) compared to resting B cells (D0) in these native culture conditions. Interestingly, cenRNA accumulation at D10 correlated with the lowest levels of *DIS3* transcripts, again suggesting a role for DIS3 in regulating these ncRNAs in physiology (Fig. 4F).

Independent of PC differentiation, we used the SSK41 cell line to decipher the role of these cenRNAs in B cells. We treated these cells for 72 h with ASOs and, similarly to primary B cells, we observed decreased proliferation (Fig. S4I, J). Inhibition of the RNA exosome activity with ERD03 also induced a strong blockade in cell division (Fig, S4K, L). We generated clones stably expressing Cas9 with doxycycline-dependent expression of guide RNAs [46]. *DIS3* genetic deletion led to similar lower proliferation compare to the wt or Cas9^+^ SSK41 control cells (Fig. S4M, N). In parallel, we transfected SSK41 wt cells with a *DIS3-T2A-GFP* plasmid, to express *DIS3* mRNA without the intron and the donor splicing site targeted by DIS3 ASOs. *DIS3* mRNA expression successfully rescued the defects induced by DIS3 ASOs, restoring proliferation levels close to those of the controls (Figs. S4O, P).

We then investigated cenRNA dynamics in SSK41 wt cells. We inhibited RNA exosome activity with ERD03, and again, we observed a substantial accumulation of cenRNAs with about a 25-fold increase (Fig. S4Q). In parallel, we stimulated SSK41 wt cells with CD40L or cytokines (IL-2, IL-4, and IL-10), and observed higher levels of cenRNAs compared to resting cells, but with a lower magnitude, about a 3-fold increase with CD40L, likely related to transcriptional activation (Fig. S4R).

Finally, we investigated cenRNA expression in the human B cell lineage from in vivo sorted populations [47]. We observed intermediate expression in naïve/memory B cells, low expression in terminally differentiated PCs from tonsil and bone marrow niches, and high cenRNA expression in GC-derived centroblasts and centrocytes (Fig. S4S), supporting a dynamic expression of cenRNAs in the human B cell lineage in vivo.

### Centromeric R-loop accumulation in DIS3-deficient cells

CenRNAs can create DNA:RNA hybrids at centromeres, a phenomenon observed in multiple species, including humans [48–51]. We hypothesize that DIS3 may regulate RNAs associated with these structures, as an accumulation of R-loops is observed in DIS3-deficient mouse activated B cells [7] and in MM cells [52]. We treated cells at D4 with ASOs and collected genomic DNA at D6 to perform R-loop identification assisted by nucleases (RIAN) experiments [53], a recently published method that offers high resolution and may be able to capture these structures at highly repetitive centromeres. We then performed qPCR analyses of centromeric R-loops on each chromosome where we identified cenRNA accumulation, and detected substantial amplification signals in cells treated with ctrl ASOs (Fig. 4G). Importantly, RNase H treatments almost totally abrogated these amplifications (Fig. 4G), as RNase H sensitivity is a critical hallmark of DNA:RNA hybrids, it demonstrated the specificity of this assay. Strikingly, DIS3 inhibition increased R-loop levels at all evaluated centromeres, while RNase H treatments abolished these amplification signals, returning them to basal level (Fig. 4G). We concluded that DIS3 is a regulator of both centromeric transcripts and centromeric R-loops, and in its absence, an important accumulation of cenRNAs and R-loops is observed at centromeres. As cenRNA and R-loop accumulation at centromeric regions is associated with genome fragility [48, 49, 54–56], they may threaten genomic stability in DIS3-deficient cells. We also evaluated chromatin accessibility at centromeres and observed a slight increase in DIS3-deficient cells (Fig. S4T), which likely reflects R-loop persistence at these regions and may contribute to the proliferation defects.

CenRNA accumulation has also been linked to the delocalization of the histone variant CENP-A from centromeres, impeding subsequent kinetochore formation and mitosis [45, 57, 58]. We evaluated the impact of cenRNA accumulation in SSK41 B cells treated with DMSO or ERD03, ctrl or DIS3 ASOs, and similarly, we observed by confocal microscopy decreased CENP-A protein intensity in the absence of RNA exosome activity (Fig. 4H, I). In ctrl cells, CENP-A proteins were clustered as sharp dots in the nuclei, while the signals were more diffuse and less intense in DIS3-deficient cells. This alteration may result from changes in CENP-A localization to the centromeres, despite comparable levels of CENP-A transcripts and protein in our cells of interest (Fig. S4U, V). These data are consistent with the altered pathway “protein localization to chromosome, centromeric region” (Fig. 3D). We hypothesized that the ratio between cenRNA transcripts and CENP-A proteins could influence CENP-A localization and subsequent proliferation. We generated multiple clones that overexpress CENP-A, but again observed proliferation defect following DIS3 depletion (Fig. S4W, X), suggesting that beyond the level of cenRNAs, R-loop accumulation may contribute to altered CENP-A localization and poor proliferation. Finally, we used siRNA to directly inhibit CENP-A during PC differentiation. In these conditions, we observed fewer PBs in CENP-A-deficient cells, demonstrating the direct role of CENP-A in ensuring B cell proliferation and subsequent PC differentiation (Fig. S4Y, Z). These results clearly demonstrate that active proliferation is required for differentiation into PCs, with CENP-A playing a critical role.

Overall, these data suggest a first level of transcriptional regulation for cenRNAs during B cell activation and differentiation that may be important for B cell physiology and adaptive immune response. The strong accumulation of cenRNAs in the absence of RNA exosome activity may reflect their post-transcriptional regulation by this complex, which fine-tunes their abundance, as well as the levels of DNA-associated RNAs at centromeres. RNA exosome activity is also necessary for proper localization of CENP-A in B cells, an essential actor of the cell cycle, likely through the degradation of cenRNAs and DNA-associated RNAs to enable CENP-A loading to centromeric chromatin. To conclude, cenRNA transcripts are dynamically expressed in the B cell lineage and their surveillance by the RNA exosome complex may be important for proliferation and associated PC differentiation.

### Genomic instability in DIS3-deficient cells

**Fig. 5:**
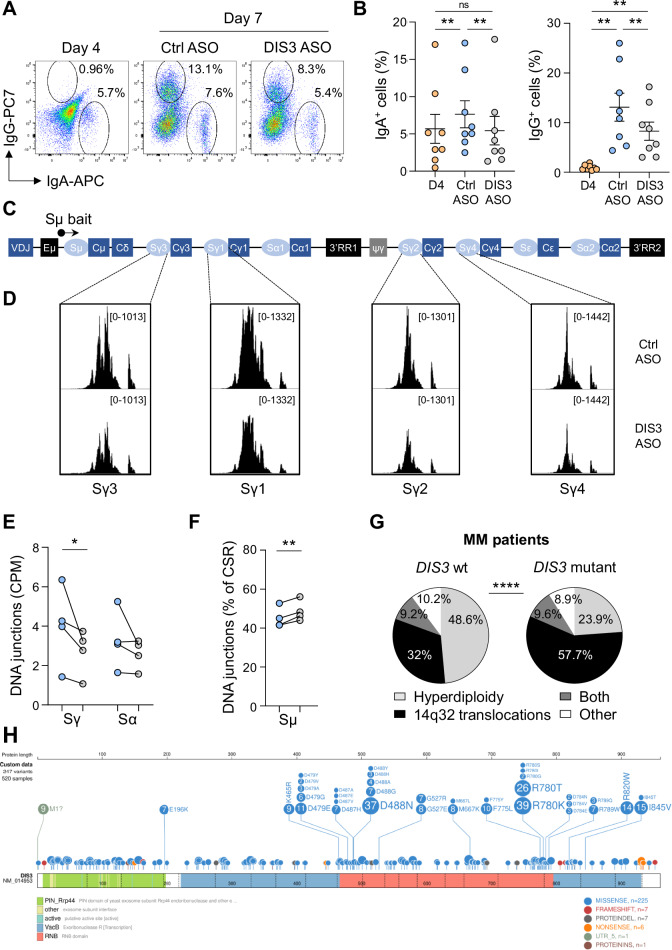
Genomic instability in DIS3-deficient cells. **A** Flow cytometry analyses of CSR. Cells were analyzed for IgA and IgG expression at D4 and D7, after ctrl or DIS3 ASO treatments. **B** Quantification of IgA^+^ and IgG^+^ cells from flow cytometry analyses. Means ± s.e.m. and individual values are shown, two-tailed paired *t*-test, 8 independent experiments, *n* = 8. **C** Schematic representation of the human *IGH* locus and the Sµ bait (not scaled). **D** Physiological CSR junctions at Sγ regions captured by LAM-HTGTS. Primary B cells were treated from D4 to D7 with ctrl or DIS3 ASOs before DNA extraction and analyses. Data are representative of 4 independent experiments. **E** Quantification of physiological CSR junctions at the Sγ and Sα regions. Individual values are shown, two-tailed ratio paired *t*-test, 4 independent experiments, *n* = 4. **F** Percentage of intra Sµ recombination. Individual values are shown, two-tailed ratio paired *t*-test, 4 independent experiments, *n* = 4. **G** Genomic instability in MM patients. Hyperdiploidy, 14q32 translocations, both or other were quantified in control (*DIS3* wt, *n* = 3077) versus *DIS3* mutant patients (*n* = 520). Two-sided Khi^2^ proportion test. **H** *DIS3* mutations in MM patients. *DIS3* mutations were analyzed (*n* = 520) and protein-coding mutations (*n* = 247) were mapped and classified

DIS3 and the RNA exosome complex are strictly necessary for efficient CSR in mice [6, 7, 59, 60]; however, their contribution to DNA recombination in humans has not been established. To answer this question, we took advantage of our differentiation model, which also features *AICDA* expression (Fig. S5A), enabling AID-mediated CSR [27]. We knocked down DIS3 expression to study CSR directly by flow cytometry and compared cells from the same donor at D4 and D7, before and after ASO treatments. In our conditions, we observed the presence of IgA^+^ cells at D4, with a slight but significant increase in ctrl cells at D7, while the frequency of IgA^+^ cells remained similar in DIS3-deficient cells (Fig. 5A, B). By contrast, we observed few IgG^+^ cells at D4 but an important accumulation at D7 in both conditions. However, DIS3-deficient cells displayed fewer class-switched IgG^+^ cells compare to the control (Fig. 5A, B). These data support an important, evolutionary conserved role of DIS3 in class switching.

In parallel, we collected genomic DNA for performing linear amplification-mediated high-throughput genome-wide sequencing (LAM-HTGTS) experiments [61] to map the unique DNA junctions at switch (S) regions. We optimized this protocol with a bait located 5’ to the switch Sµ region (Sµ bait, Fig. 5C). The quality and quantity of each library were validated before deep sequencing on Illumina Mi-seq with paired-ended 2×300 bp sequencing technology.

We aligned our data to the T2T-CHM13 genome and obtained several thousand CSR junctions from multiple donors. We first visualized the reads corresponding to these DNA junctions and observed a similar qualitative distribution along the *IGH* locus in each condition, with signals overlapping all the S regions (Fig. S5B). However, we noticed fewer DNA junctions at all Sγ regions in DIS3-deficient conditions (Fig. 5D). We quantified the absolute number of recombination events and confirmed lower CSR to Sγ, and to a lesser extent, lower CSR to Sα, associated with DIS3 deficiency (Fig. 5E and S5C). We then quantified the relative distribution of CSR junctions inside the *IGH* locus and observed a similar trend, with relatively fewer junctions in Sγ compared to Sα regions (Fig. S5D). In this distribution, we also noted more intra-Sµ recombination in DIS3-deficient cells, a hallmark of unsuccessful CSR where DNA DSBs inside the Sµ region were repaired together instead of being joined to a distal partner for productive CSR (Figs. 5F and S5D). We calculated the lengths of microhomologies in DNA junctions for each S region. We noticed the presence of junctions containing 5 or more base pairs (bp) of microhomology at the Sγ regions, this percentage increased for Sµ, and even more for Sα, in agreement with recent observations [62], while DIS3 deficiency did not alter this distribution in our conditions (Fig. S5E). We also calculated the percentage of insertions in S regions and did not observe significant changes (Fig. S5F). Since performed with total B cells that include IgG and IgA switched mBCs, these experiments were initiated with preexisting junctions, which may partially occult the impact of DIS3 loss on newly generated CSR junctions accumulated during the in vitro stimulations, especially for IgA CSR (Fig. 5A, B). However, our results clearly demonstrate the involvement of DIS3 in physiological CSR to IgG and IgA in humans, while the increased aberrant intra-Sµ recombination likely represent a sink for those Sµ breaks failing to find a downstream CSR partner.

DIS3 deficiency has been linked to genomic instability in mouse activated B cells [7] and MM cell lines [52]. GSEA analyses revealed an alteration in DNA repair pathways; we, therefore, aimed to evaluate the impact of DIS3 on genomic instability in human B cells. As a marker of genetic instability, we quantified phospho-γH2AX by confocal microscopy in SSK41 cells treated with ERD03 and observed a ~2-fold increase of this mark associated with DNA DSBs (Fig. S5G, H).

Finally, in our large cohort of newly diagnosed MM patients, we observed that patients with *DIS3* mutations displayed a substantial increase in 14q32 translocations, about 80% higher than control patients (*DIS3* wt) (Fig. 5G). This over-representation of pathological recombination was observed for most of the translocations involving the *IGH* locus, including t(4;14), t(11;14), t(14;16), and t(14;20), suggesting a general mechanism of increased genomic instability in *DIS3*-mutated patients (Fig. S5I). Other genetic alterations were associated with *DIS3* mutations, including increased frequency of 1q gain and 13q deletion, but a reduced incidence of hyperdiploidy, including trisomies 5 and 21, 17p deletion, *NRAS* and *KRAS* mutations (Fig. S5I).

We further analyzed the spectrum of *DIS3* mutations in these patients by targeted sequencing of the *DIS3* gene (Fig. 5H). Among the 520 patients with *DIS3* mutations, we identified 273 non-coding mutations that may impact *DIS3* transcript stability, splicing, processing, translation, and ultimately DIS3 protein abundance and functions. We also found 247 protein-coding mutations, the majority of which were missense variants (91%), likely impacting DIS3 enzymatic activity. A minority of mutations included frameshift and deletion (2.8% each), nonsense mutations (2.4%), and rare alterations in the 5’ UTR (potentially disrupting the initiation codon) or insertion (0.4% each). We observed mutation hotspots at positions recurrently mutated in MM, corresponding to D479, D488, and R780, all located in the RNB domain, which is critical for DIS3 exoribonuclease activity [20]. Additional mutations were distributed across other regions of the protein, including the PIN domain which mediates endoribonuclease activity of DIS3, and sites potentially affecting the conformational stability of DIS3, RNA binding, interactions with RNA exosome subunits or cofactors, and ultimately compromising nuclear RNA exosome catalytic properties (Fig. 5H). Most of these mutations were C to T transitions (~80%), a hallmark of AID-mediated mutagenesis (Fig. S5J).

These data confirm the crucial role of DIS3 in supporting the DNA recombination machinery during physiological CSR, both in mice and now in humans. In the absence of DIS3, aberrant intra-Sµ recombination and genomic instability were increased. Critically, patients with *DIS3* mutations exhibit significantly more 14q32 translocations than control patients, suggesting a role for DIS3 as a tumor suppressor, particularly for preventing aberrant pathological DNA recombination.

## Discussion

Mutations of the *DIS3* gene were first described in MM patients by Chapman et al. [19] and have since been corroborated by numerous studies. Additionally, loss of heterozygosity of the *DIS3* gene has been consistently documented [15–17]. Together, these findings highlight the increasing recognition of *DIS3* alterations and their detrimental impact on the survival of MM patients [18, 22]. Mouse models have demonstrated the essential role of DIS3 during early B cell development and V(D)J recombination [5], as well as in activated B cells undergoing CSR and SHM [7]. These investigations underscore the importance of DIS3 in processing R-loops and ncRNAs expressed at Ig genes, thereby ensuring access for the enzymes recombination-activating gene (RAG) and AID to mediate efficient recombination [3].

Additional models have been developed to further investigate DIS3-dependent mechanisms involved in MM. In mice, early deletion of the *Dis3* gene in hematopoietic cells was revealed to be insufficient for myelomagenesis [63]. Studies in MM cell lines have reinforced the critical role of DIS3 in processing R-loops to prevent genomic instability [52], and in supporting cell proliferation within a pathological context [64]. However, an effective model to elucidate the role of DIS3 in PC pathophysiology has not been firmly established. In this study, we developed a DIS3 ASO strategy, which proved to be a highly efficient tool for knocking down DIS3 expression during PC differentiation. The partial depletion of the DIS3 protein, attributed to its long half-life and the stability of the RNA exosome, mirrors the partial LOF observed in *DIS3* mutant MM patients. Notably, complete knockout of the *Dis3* gene is lethal in mice [23]. However, DIS3 ASOs only decrease *DIS3* expression, while *DIS3* mutations in patients may generate dominant-negative proteins, which may accumulate on canonical DIS3 substrates and preserve the ability to recruit exosome partners. These partners include AID [59], with critical implications for mutagenesis and genome stability when the exosome is recruited to chromatin-associated RNAs.

The name *Dis3* originates from its role in chromosome *dis*junction, as it was originally identified to be necessary for mitotic chromosome segregation in the fission yeast *Schizosaccharomyces pombe* [65]. DIS3 is actually implicated in cell proliferation across various organisms, including *Drosophila* [66] and humans [20]. Single-cell RNA sequencing has shown elevated *DIS3* expression in actively cycling PC precursors [29, 30], and we demonstrate that DIS3 is necessary for the proliferation of both mouse [7] and now human activated B cells. DIS3-deficient cells accumulated as CFSE^high^ and exhibited an altered transcriptome, characterized by reduced expression of genes involved in cell cycle progression, such as the key cyclin A2. Additionally, other genes associated with proliferation in both normal and cancerous cells, including *RPLC34*, *PARM1*, and *CHPF*, showed decreased expression associated with DIS3 deficiency. In mice, *Dis3* is also essential for B cell proliferation and PC differentiation [33, 34], suggesting evolutionary conserved functions.

DIS3 depletion reduced switched Ig production, likely reflecting a failure to differentiate into antibody-secreting cells and/or CSR defects. Decreased Ig secretion may also be due, at least in part, to disruptions of the UPR pathway, as illustrated by decreased expression of genes such as *CANX*. Mutations in genes involved in the UPR, particularly *XBP1*, have been documented in MM [19], with Xbp1s-deficient tumor cells acquiring resistance to proteasome inhibitors [67]. Further investigations are necessary to better understand the impact of DIS3 on this critical function in the pathophysiology of MM.

We previously noted a higher frequency of Igλ^+^ cells in mice with early deletion of *Dis3* [5], which we attributed to defective recombination at the *Igk* locus. However, this likely does not apply in our system, which uses mature B cells and where the increased presence of *IGL* transcripts and Igλ^+^ cells likely results from a more favorable selection process in this context. The *IGK* locus, due to its complex organization, may be more affected by DIS3 depletion than the *IGL* locus, leading to disrupted *IGK* expression levels, lowering BCR assembly, tonic signal, and B cell proliferation/survival of Igκ^+^ cells. This observation is relevant as patients with *DIS3* mutations also exhibit a higher incidence of IGL myeloma, possibly resulting from a selection process. Again, additional investigations are crucial to uncover the mechanisms underlying this phenomenon.

The substrates of the RNA exosome have been well characterized and include eRNAs, antisense promoter-associated RNAs, and other ncRNAs [2]. In *Schizosaccharomyces pombe*, which has a specific centromere organization, cenRNAs are also sensitive to DIS3 activity [68]. Our data suggest that cenRNAs are DIS3 substrates also in mammal cells, with important implications for cellular homeostasis. In mammals, centromeres are composed of repeated α-satellites that form heterochromatin and were thus considered transcriptionally inactive. It is now well established that centromeres are actively transcribed and that cenRNAs are crucial for mitosis [43, 69, 70]. These cenRNAs regulate kinetochore assembly, notably through interactions with the histone variant CENP-A [45]. Given their crucial role, cenRNAs must be tightly regulated, both at the transcriptional and post-transcriptional levels. Recently, the post-transcriptional regulator Rio1 was shown to maintain low levels of cenRNA for efficient kinetochore assembly and mitosis [71], demonstrating that excessive levels of cenRNAs impair proliferation. Similarly, we demonstrate that DIS3 maintains physiological levels of cenRNA in activated B cells for efficient proliferation and associated PC differentiation. Furthermore, in the absence of RNA exosome activity, cenRNA and centromeric R-loops accumulate, while CENP-A localization is altered, likely contributing to proliferation defects, as CENP-A is globally necessary for cell proliferation [72, 73], and we observed a direct impact of CENP-A knockdown on B cell proliferation and PC differentiation.

CenRNA and centromeric R-loop accumulation can also lead to genomic instability, as observed in yeast [49, 71] and cancerous cells [50, 54, 56, 74, 75]. Inversely, DNA DSBs at centromeres induce cenRNA transcription [76]. It is therefore possible that cenRNA accumulation at centromeres contributes to increased DSBs in these regions, or conversely, that the accumulation of DSBs triggers cenRNA overexpression, presenting a complex chicken-and-egg situation. CenRNAs interact with various RNA-binding proteins, including DNA repair proteins such as MRE11 [77], an essential actor of the classical non-homologous end-joining (NHEJ) in B cells [78]. CenRNA accumulation may thus trap MRE11 or other proteins, contributing to poor CSR and eventually to genomic instability. Centromeric R-loop accumulation may also directly contribute to genomic instability in DIS3-deficient cells.

The increased proportion of patients with IgA myeloma may be attributed to alternative DNA repair mechanisms at Sα, utilizing both classical NHEJ and robust alternative end joining (aEJ), while repair at other S regions is more reliant on classical NHEJ [62]. In *Dis3* knockout mouse B cells, increased lengths of microhomologies at DNA junctions were observed [7] as a hallmark of aEJ, again suggesting that classical NHEJ is more sensitive to DIS3 depletion, and aEJ is more efficient in the absence of DIS3. DNA repair mechanisms are cell cycle-dependent [79], with CSR lesions processed during the G1 phase [80]. Perturbations in cell cycle progression may contribute to lower CSR efficiency, affecting DNA repair and redirecting toward pathological recombination and translocations.

3D synapsis at the mouse *Igh* locus is necessary to bring S regions into a recombination factory, so they can be repaired together in a productive deletional CSR event. This proximity is dependent on the interactions between the Eµ and 3’RR enhancers [81] and relies on cohesin-mediated loop extrusion [82]. In mice, DIS3 deficiency induces eRNA accumulation at the 3’RR and alters the *Igh* locus organization, decreasing CSR and eventually facilitating translocations [7]. Similarly, here, we observe eRNA accumulation at the human 3’RRs that may compromise the *IGH* synapsis, associated with increased intra-Sµ recombination, which likely results from altered *IGH* locus organization where Sµ breaks fail to find a long-range recombination partner. Defective *IGH* 3D organization may also contribute to the substantial increase of 14q32 translocations observed in patients with *DIS3* mutations.

Our comprehensive analysis of *DIS3* mutations in newly diagnosed MM patients reveals a complex mutational landscape, including a substantial burden of non-coding alterations. These non-coding mutations may affect transcript stability, processing, and translation, thereby influencing DIS3 expression and function. The protein-coding mutations were mostly missense variants, consistent with previous reports [17, 19, 22], while other alterations were observed at lower frequencies, including frameshift, deletion, and nonsense mutations. Disruption of the 5’UTR was observed in one patient, as well as one insertion. These alterations were mainly localized in the RNB domain, with known hotspots at D479, D488, and R780, likely decreasing 3′ to 5′ exonucleolytic degradation of RNAs. We also observed mutations within the PIN domain, which contributes to the endoribonuclease activity and may facilitate the resolution of structured or circular RNAs [83, 84]. Globally, *DIS3* alterations may alter protein folding, RNA binding interfaces, interactions with exosome subunits and cofactors, ultimately leading to perturbation in RNA processing and degradation, including DNA-associated RNAs. These alterations may cooperate with other genomic events to drive disease progression.

MM is considered a post-GC PC malignancy [85], characterized by a high level of genomic instability [26]. This pathology is preceded by pre-malignant stages, such as MGUS and SMM that can eventually progress to fatal MM. Primary genetic events such as *IGH* translocations and hyperdiploidy, which are usually mutually exclusive, initiate the disease while secondary genetic events such as copy number abnormalities and somatic mutations are implicated in the progression toward MM. It is still unclear how DIS3 LOF is implicated in MM initiation and/or progression. *DIS3* alterations may occur during B cell expansion in GCs, altering the proliferation process and overexposing cells to the mutagenic action of AID, inducing genomic instability, especially through *IGH* translocations. In agreement, we and others [17, 22] observed a significant increase in translocations in *DIS3* mutant patients. These translocations likely provide a selective advantage, enabling *IGH* 3’RR super-enhancers to drive the expression of oncogenes. In contrast, the development of hyperdiploidy appears to rely on distinct mechanisms and is a gradual process that unfolds over several years [86]. In our cohort, *DIS3* mutations were dominated by C to T transitions, consistent with AID-mediated mutagenesis. Altogether, these data strongly suggest that DIS3 deficiency causally leads to increased genomic instability and that *DIS3* may act as a tumor suppressor gene in this cancer. This is further emphasized by the fact that patients with germline variants of *DIS3* are predisposed to develop a familial MM [21]. DIS3 deficiency may also facilitate secondary genetic events in MGUS [24] and SMM [25] and thus contribute to disease progression toward MM. Finally, *DIS3* alterations in MM cells may further increase genomic instability, contributing to poor prognosis.

Another important issue for PCs is related to their fate, as they can differentiate either into short-lived or long-lived (LLPCs). While it was long assumed that extrafollicular PCs were short-lived and that only GC responses could yield LLPCs, recent studies have acknowledged the existence of GC-independent LLPCs and shown that PC survival can be determined secondarily, relying on factors provided by the bone marrow PC niche [87]. The impact of DIS3 on this short versus long lived remains a relevant question for myelomagenesis to answer.

Overall, this study provides a new insight into the molecular mechanisms involving DIS3 that are relevant to human PC pathophysiology (Fig. S5K).

## Materials and methods

All reagent detailed information and primers used for this study are listed in Table 3.

### Human plasma cell differentiation

Total B cells were purified from healthy donor buffy coats obtained from the “Etablissement Français du Sang” (EFS) Bretagne. All volunteers were recruited in accordance with the national French guidelines of the EFS after providing informed consent for the use of their blood cells in research. Buffy coats were subjected to red blood cell lysis (DAKO EasyLyse™, Agilent) and pre-purification phenotype analysis by flow cytometry based on CD44, CD19, and CD27 expression. CD19^+^ total B cells were purified using CD19 MicroBeads with a MultiMACS™ Cell24 Separator (Miltenyi Biotec), according to the manufacturer’s instructions. Purity was verified by flow cytometry based on CD20 and CD45 expression (usually >90%). On day 0 after purification, cells were labeled with CFSE (Invitrogen) and seeded in 24-well plates at a density of 0.75 × 10^6^ cells/mL in RPMI 1640 Glutamax (Gibco) supplemented with 10% fetal bovine serum (Eurobio scientific), sodium pyruvate (ThermoFisher Scientific), non-essential amino acids (ThermoFisher Scientific), and penicillin/streptomycin (10^4^ U/mL, ThermoFisher Scientific). Cells were stimulated with an activation cocktail consisting of 100 ng/mL of CD40L (Immunex), 50 U/mL of IL-2 (R&D Biosystems), 1 µg/mL of CpG ODN2006 (Miltenyi Biotec), and 2 µg/mL of anti-IgA/IgM/IgG BCR (Jackson Immunoresearch). When necessary, cells were treated on day 1 (24 h after purification) with ASOs, ERD03, QVD-OPH, or STLC. On day 2, 5 ng/µL of IL-10 (R&D Biosystems) was added to boost B cell activation. On day 4, cells were pooled, washed with PBS, and seeded at 0.5 × 10^6^ cells/mL in 24-well plates with fresh RPMI. Cells were stimulated with a differentiation cytokine cocktail consisting of 50 U/mL of IL-2, 5 ng/mL of IL-4 (R&D Biosystems), and 12.5 ng/mL of IL-10, and treated with appropriate concentrations of ASOs, ERD03, QVD-OPH, or STLC. CFSE dilution and CD38 expression were analyzed by flow cytometry. On day 7, cells were pooled, and differentiation was analyzed by flow cytometry based on CFSE dilution and CD38 expression. For the final step of differentiation, cells were collected on day 7 and seeded at 0.5 × 10^6^ cells/mL with fresh RPMI in 24-well plates. Cells were stimulated with a PC differentiation cytokine cocktail consisting of 50 U/mL of IL-2, 12.5 ng/mL of IL-10, 40 ng/mL of IL-6 (Peprotech), and 250 U/mL of IFN-α2b (PBL assay science). On day 10, cells were pooled and analyzed by flow cytometry for CD38 and CD138 expression. Samples were collected throughout the differentiation protocol for DNA, RNA, and protein analyses.

### Mouse plasma cell differentiation

Spleens were collected from heterozygous (C57BL/6J crossed with 129/SvJ mice, Janvier Labs) mice aged 6 to 8 weeks. Spleens were flushed and subjected to red blood cell lysis with ACK buffer. Resting B cells were isolated using CD43 magnetic beads and LS columns on a QuadroMACS™ separator (Miltenyi Biotec). Cells were cultured at 1 × 10^6^ cells/mL in RPMI supplemented with 10% fetal bovine serum, sodium pyruvate, non-essential amino acids, and penicillin/streptomycin (10^4^ U/mL), stimulated with 1 ng/µL of LPS (Invivogen), and treated with 4 µM ASOs for 72 h. Cells were analyzed by flow cytometry based on CD138 (BD Biosciences) and B220 (BD Biosciences) expression (Table 3).

### Cell line

SSK41 is a B lymphocyte cell line isolated from a patient with follicular lymphoma [88]. SSK41 cells were kindly provided by Prof. Francesco Bertoni (Oncology Institute of Southern Switzerland). Cells were grown in RPMI + Glutamax supplemented with 10% fetal bovine serum, sodium pyruvate, non-essential amino acids, and penicillin/streptomycin (10^4^ U/mL). Cells were tested negative for mycoplasma.

### ASO treatments

“Vivo-Morpholino” ASOs were purchased from Gene Tools and reconstituted in nuclease-free water (Invitrogen) at 500 µM following the manufacturer’s instructions. We designed the DIS3 ASO sequences; the control ASO is an irrelevant sequence from the manufacturer. ASOs were directly added to the cultures at the indicated concentrations; 1.25 µM (D1 to D4), 2.75 µM (D4 to D7) and 3.5 µM (D7 to D10) for human cells, and 4 µM for mouse cells, below toxicity effects.

### Chemicals

Chemicals were purchased from MedChemExpress or Sigma-Aldrich. QVD-OPH was used at 10 µM as a pan-caspase inhibitor. STLC was used at a concentration of 6 µM to block cells in mitosis. ERD03 was reconstituted in DMSO at 100 mM and used at 200 µM (D1 to D4), 300 µM (D4 to D7) or 400 µM (D7 to D10) as an RNA exosome inhibitor.

### Transfections

SSK41 B cells were transfected with Amaxa kit V (VCA-1003, program X001). For DIS3 overexpression, the *DIS3* mRNA transcript (variant 1) was synthesized by GeneCust and cloned into pcDNA3.1 expression vector. The cassette includes the *DIS3* mRNA, a sequence corresponding to T2A for self-cleavage, followed by GFP as a reporter. We transfected SSK41 cells and studied bulk populations, allowing direct comparison of GFP^-^ and GFP^+^ cells in the same culture. For conditional deletion of the *DIS3* gene, we used a dual reporter system [46]. Briefly, Cas9-mCherry SSK41 cell lines were generated by lentiviral transduction, sorted by flow cytometry, and multiple clones were generated. Secondly, we used doxycycline (Dox)-inducible single-guide RNA (sgRNA) expression system, consisting of an sgRNA cassette under the control of a tetracycline response element, and a ubiquitin promoter-driven tetracycline repressor co-expressed with GFP via a T2A self-cleaving peptide. SSK41 Cas9^+^ cells were transduced by lentivirus, double-positive cells were sorted and multiple clones were generated. Induction with Dox enabled regulated sgRNA expression in GFP-positive cells. For competition assays, we mixed wt SSK41 with double-positive cells, or Cas9^+^ SSK41 with double positive cells, and treated cells or not with Dox for 10 days to allow sufficient time for deleting both alleles. We followed the cellular ratios by flow cytometry. For CENP-A overexpression, we used the CENP-A-GFP plasmid [89] (Addgene #117803), GFP^+^ cells were sorted at D4, and multiple clones were amplified. Primary B cells were transfected at day 2 with ctrl (Horizon Discovery, D-001810-10-05) or CENP-A (Horizon Discovery, L-003249-00-0005) siRNAs as previously described [90].

### Flow cytometry and cell sorting

For CSFE staining, cells were labeled on day 0 using CellTrace™ CFSE or CellTrace™ Far Red (Invitrogen) according to the manufacturer’s instructions. For surface markers, cells were washed in PBS and incubated with fluorescent antibodies (Table 3) for 30 min at 4 °C in FACS buffer (PBS 3% fetal bovine serum), washed in PBS and resuspended in FACS buffer. DAPI was added before analysis. Data were acquired on a Beckman Coulter CytoFlex flow cytometer; cells were analyzed as live single cells using the FlowJo software (version 10.10.0). aBC, prePB, and PB cell populations were sorted based on CFSE dilution and CD38 expression using a FACSAria™ Fusion cytometer (BD Biosciences).

### RNA extraction and reverse-transcription

Cells were lysed in TRIzol™ solution (Invitrogen) and total RNA was extracted following manufacturer’s instructions. RNA pellets were resuspended in DNase/RNase-free distilled water (Invitrogen). RNA concentration and purity were quantified by spectrophotometry (DeNovix) and quality (RIN) was determined using the High Sensitivity RNA ScreenTape kit with a TapeStation 4200 (Agilent). Reverse transcription was performed using the Superscript II reverse-transcriptase (Invitrogen) with random primers following the manufacturer’s instructions.

### PCR and quantitative PCR (qPCR)

Exon skipping was evaluated by PCR using the Taq’Ozyme Purple Mix 2 (Ozyme). PCR products were analyzed by electrophoresis on 2% agarose gels. qPCR analyses were performed with 4 to 40 ng of cDNA using the Power SYBR™ Green PCR Master Mix (Applied Biosystems) according to the manufacturer’s instructions with a QuantStudio 3 (Applied Biosystems). Data were analyzed using the delta Ct method using *ABL1* and *HPRT1* as housekeeping genes for normalization. Primer sequences are listed in Table 3.

### RNA-sequencing

RNA-sequencing was performed by Genewiz (Azenta Life Sciences) using the Strand-specific RNA-seq bundle after rRNA depletion with 100 million reads per sample. ERCC spike-in were included as controls.

### Western blot

Protein extracts were produced from cell pellets using RIPA buffer (Pierce), according to the manufacturer’s instructions. Samples were sonicated 2–3 times for 5 s at 25% intensity. Protein concentration was measured by Bradford assay (BioRad). Proteins were separated by electrophoresis on 4–12% NuPAGE mini gels (Invitrogen) and transferred onto PVDF membranes using the iBlot2 system (Invitrogen). Membranes were saturated with TBS-T + 5% non-fat dried milk and incubated overnight at 4 °C with actin, DIS3, or CENP-A primary antibodies (Table 3). The next day, membranes were washed and incubated for 1 h with HRP-conjugated secondary antibodies (BioLegend) at room temperature. Membranes were washed again and subjected to substrate revelation using SuperSignal reagent (ThermoScientific). Images were obtained with a GBox system (Syngene) and analyzed with Fiji/ImageJ (v2.14).

### ELISA

Culture supernatants were collected by centrifugation and stored at −20 °C. For ELISA assays, plates were coated overnight with primary antibodies specific to human IgM, IgG or IgA (Southern Biotech) (Table 3). Plates were loaded with 30 µL of culture supernatant per well for 2 h at room temperature, washed with PBS-T, and incubated with HRP-conjugated secondary antibodies diluted in PBS + 3% BSA, according to the manufacturer’s protocol. Plates were revealed using ρ-Nitrophenyl phosphate tablets (Sigma-Aldrich), coloration was stopped by the addition of 3 N NaOH, and absorbance was read at 405 nm using a Synergy H1 plate reader (Agilent). Data were analyzed based on standard Ig solutions.

### Microscopy

For CENP-A analysis, 6 × 10^5^ cells were washed with PBS, resuspended in PBS and subjected to hypotonic shock by addition of 400 µL of pre-heated 75 mM KCl. Cells were adsorbed on microscopy slides using a cytospin and fixed for 10 minutes with 4% paraformaldehyde. Slides were washed and blocked for 45 minutes at room temperature with blocking buffer (PBS, 0.1% Triton, 20% BSA, 2% donkey/goat serum). Cells were immunolabelled for 1 h at room temperature with anti-CENP-A antibody (1:100, Invitrogen) **(**Table 3**)** diluted in buffer (PBS, 20% BSA, 2% goat/donkey serum) and washed 4 times with PBS + 0.1% saponin. Slides were then incubated for 1 h with a secondary antibody (1:1,000, Jackson Immunoresearch), washed 4 times with PBS + 0.1% saponin and mounted with Fluoromount G + Sytox Green (BioLegend, Invitrogen). For γH2AX analysis, 6 × 10^5^ cells were washed with PBS, resuspended in 100 µL of PBS and dropped on Poly-Lysine-coated microscopy slides. Cells were incubated on the slides at 37 °C for 20 min and fixed for 10 min with 4% paraformaldehyde. Slides were blocked for 45 min at RT with blocking buffer and incubated overnight with anti-phospho-γH2AX antibody diluted in buffer (1:250, Cell Signaling Technology) **(**Table 3**)**. Slides were then processed as described above. All images were obtained with an SP5 confocal microscope (Leica) and analyzed using Fiji/ImageJ (v2.14).

### LAM-HTGTS

Differentiating cells were treated between D4 and D7 with 3 µM of ctrl or DIS3 ASOs and harvested at D7. Cells were lysed in proteinase K buffer with 10 µg of proteinase K (Cell Signaling Technology) at 56 °C overnight. Genomic DNA was extracted by ethanol precipitation and resuspended in TE buffer for LAM-HTGTS library preparation, as previously described [61]. Fifteen to 40 µg of genomic DNA was sonicated to generate 500 to 1500 bp fragments using an Epishear Probe Sonicator (Active Motif, 3 cycles, 25% energy output, 5 s working time, 30 s resting). The Q5 polymerase (New England Biolabs) was used for the amplification steps. Primers used for each step are described in Table 3. Library purification was performed using Select a size DNA Clean & Concentrator kit (Zymo Research) with a cut-off of 300 nt. Library size and quality were assessed using the TapeStation 4200 with D1000 High Sensitivity Kit (Agilent). Libraries were quantified using the KAPA library quantification kit (Roche). Final libraries were pooled into an equimolar mix at 4 nM and denatured with NaOH, according to the procedure recommended by Illumina. Libraries were sequenced on an Illumina MiSeq using the MiSeq Reagent Kit V3 (600 cycles) (Illumina).

### R-loop identification assisted by nucleases (RIAN)

RIAN experiments were performed as previously described [53]. Briefly, DNA-associated RNAs were enriched from genomic DNA extracted with Qiagen DNeasy blood and tissue kit (69504). DNA was digested with restriction enzymes (AluI, MboI, MseI, DdeI, NEB) and nucleases that only preserve R-loops (nuclease P1, T5 exonuclease, lambda exonuclease, NEB). This reaction was split in two after one hour at 37 °C, RNase H (NEB) was added to one tube followed by one hour of incubation at 37 °C. qPCR were performed using the same oligonucleotides as RT-qPCR for cenRNAs and were normalized to the input DNA.

### Assay for transposase-accessible chromatin (ATAC)

ATAC experiments were performed as previously described [91] on differentiating cells at day 6, 48 h after ASO treatments. Accessible DNA was quantified by qPCR, using a negative (*MYT1*) and a positive control (*ACTIN*).

### DNA *IGH* library preparation

*IGH* VDJ regions were amplified and sequenced from 500 ng of total genomic DNA. First, genomic DNA was amplified using BIOMED-2 primers [42]. In a second PCR step, Illumina barcodes were incorporated. Primer sequences are listed in Table 3. Sequencing was performed on a MiSeq system using the MiSeq Reagent Kit v3 (600 cycles) (Illumina, San Diego, CA, USA).

Briefly, library preparation began with PCR amplification of 500 ng of DNA using AmpliTaq Gold (Thermo Fisher) and BIOMED-2 primers modified for Illumina sequencing. The reaction was carried out in a final volume of 50 µL. PCR cycling conditions were: 7 min at 95 °C, followed by 34 cycles of 30 s at 95 °C, 1 min at 60 °C, and 1 min at 72 °C, with a final extension of 10 min at 72 °C. Illumina adapter and index sequences were then added via a second PCR. In this step, 6 µL of the first PCR product was re-amplified using Phusion DNA polymerase (New England Biolabs) in a final volume of 25 µL. PCR cycling conditions were: 30 s at 98 °C, followed by 12 cycles of 10 s at 98 °C, 30 s at 62 °C, and 30 s at 72 °C, with a final extension of 5 min at 72 °C. PCR products were purified using 1.8× volume of MagPrep purification beads (Merck) and eluted in 30 µL of elution buffer. The final library was prepared by pooling 20 µL from each sample, concentrating the pool using 1.8× volume of MagPrep beads, and eluting in 40 µL of elution buffer. The pooled library was size-selected on a PippinHT system (Ozyme) using a 1.5% agarose cassette to recover fragments in the 400–600 bp range. Final quantification was performed on an Agilent TapeStation using a D1000 ScreenTape. The library was sequenced on a MiSeq using the v3 600-cycle kit, loaded at 14 pM with 30% PhiX control.

### Bioinformatic analyses

#### Databases

*DIS3* isoform data were extracted from NCBI (https://www.ncbi.nlm.nih.gov/gene/22894).

### Single-cell RNA-sequencing

Data were obtained from [29] for in vitro PC differentiation and from [30] for in vivo PC differentiation.

### RNA-sequencing analyses

RNA sequencing data was processed using the nf-core/rnaseq pipeline [92] v3.13.2. Adapter trimming was carried out with Trim Galore (v0.6.7) and Cutadapt [93] (v3.4) using default parameters. Quality control was performed using FastQC (v0.12.1).

### Analyses on the human genome Hg38

Within the nf-core/rnaseq pipeline, reads were aligned to the Hg38_R90 genome using STAR [94] (v2.7.9a), and transcripts were quantified using Salmon [95] (v1.10.1), referencing GCA_000001405.25 genome annotation from NCBI. Genes with an average of fewer than 1 read per sample were prefiltered prior to differential expression analysis. The analysis was performed using DESeq2 [96] (v1.42.1), with filtering of low-expressed genes using HTSFilter [97] (v1.42.0) and multiple test correction using the false discovery rate method. GSEA was conducted using the clusterProfiler package [98] (v4.10.1) with the following parameters: ont = “BP”, keyType = “ENSEMBL”, minGSSize = 5, maxGSSize = 800, pvalueCutoff = 0.1, OrgDb = “org.Hs.eg.db”, pAdjustMethod = “fdr”.

### Analyses on the human genome Telomere-to-Telomere (T2T)

Reads were aligned to the T2T genome (GCF_009914755.1_T2T-CHM13v2.0) using BWA (v0.7.17). File indexing was performed with Samtools [99] (v1.15). Centromere coverage was determined using Samtools (v1.15), and RPKM values were calculated. Repeated elements were quantified using featureCounts [100] from the Subread package (v1.6.0), based on the RepeatMasker annotation of the T2T genome available via the UCSC Table Browser (table ID: “hub_3671779_t2tRepeatMasker”). RPKM values were then calculated, and statistical significance between conditions was assessed using a two-tailed t-test (stats.ttest_ind) implemented in SciPy [101] (v1.14.1). Aligned BAM files were also converted to BigWig format using bamCoverage from deeptools [102] (v3.4.2) with the following parameters: --normalizeUsing CPM and -bs 1. BigWig files were merged using ucsc-bigWigMerge for visualization in IGV [103].

### Transcriptome repertoire analyses

TRUST4 [40] (v1.0.5) was used to reconstruct B cell receptor sequences from RNA-sequencing data.

### DNA repertoire analyses

Paired-end reads were merged using FLASH software [104], including the concatenation of non-overlapping read pairs. IMGT/HighV-QUEST [105] was used for sequence alignment against the human IG reference database no. 202506-1. Only productive CDR-H3 sequences were retained and grouped into clonotypes using the hierarchicalClones function from the Scoper toolkit [106].

For each repertoire, Hill diversity profiles [107] were generated, and the median somatic hypermutation frequency was calculated.

### LAM-HTGTS analyses

LAM-HTGTS data analysis was performed as previously described [61]. All sequence alignments were done with the T2T genome. Analysis of junction structure was performed with the CSReport tool [108]. Data from four independent donors were analyzed individually and then pooled for global analyses.

### *DIS3* mutation analyses

Samples were sequenced with a previously published panel [109] on an Illumina NextSeq 500 sequencing machine with a median 200× depth. This panel covers the entire *DIS3, TP53, NRAS, KRAS, BRAF, FAM46C, ATM, ATR, MYC, TRAF3, BIRC2, BIRC3, CYLD, IRF4, CRBN* sequences. After demultiplexing, reads were trimmed with fastp (https://github.com/OpenGene/fastp) and Agilent’s software trimmer (https://explore.agilent.com/AGeNT-Software-Download-Form-TY) according to Agilent’s recommendations. Trimmed reads were aligned with bwa-mem and sorted and indexed with samtools (https://github.com/samtools/samtools). Duplicated reads were marked using Agilent’s software creak. The resulting bams were then processed with the gatk best practice pipeline as of version 3.6 (not documented anymore) (https://gatk.broadinstitute.org/), using the following steps: RealignerTargetCreator, IndelRealigner, picard FixMateInformation (https://broadinstitute.github.io/picard/), BaseRecalibrator and finally PrintReads. MuTect2 was used to call small variants, using the --normal_panel argument to mark known germline variants with our in-house database consisting of a large cohort of normal samples.

Resulting vcf files were annotated with SnpEff and SnpSift (https://pcingola.github.io/SnpEff/) against clinvar, ExAC, cosmic, dbsnp and gnomad and filtered with the following criterias : variant effect is not one of intron_variant, synonymous_variant, intergenic_region, interaction, AD ≥ 20 and AF ≥ 10%, variant effect is HIGH or MODERATE, variant is not observed in the panel of normals. Data were visualized using Mutation Annotation Format tools (maftools), R/Bioconductor package (https://www.r-project.org/), accessed on 11 June 2022 (R version 4.4). Mutations’ lolliplot was obtained using ProteinPaint tool [110].

### Data visualization

NGS data were converted as bed files and visualized with the Integrative Genomics Viewer (IGV, version 2.17.4).

### Statistical analyses

Statistics were performed using the GraphPad Prism software (version 8). Statistical analyses were performed using two-tailed paired *t*-tests or other appropriate statistical tests, as indicated in the figure legends. *P*-values were considered as significant when inferior to 0.05 (*), 0.01 (**), 0.001 (***), and 0.0001 (****).

## Supplementary information

manuscript unmarked

Source figure

Supplementary Figure S1

Supplementary Figure S2

Supplementary Figure S3

Supplementary Figure S4

Supplementary Figure S5

Supplementary table 1

Supplementary table 2

Supplementary table 3

## Data Availability

Sequencing data have been uploaded to the European Genome-phenome Archive (https://ega-archive.org), under the accession numbers EGAD50000001243 (for RNA-sequencing data), EGAD50000001242 (for LAM-HTGTS data), and EGAD50000001601 (for repertoire data).
