# Supplementary figures and images for: DIS3 licenses B cells for plasma cell differentiation in humans
Source: Cell Mol Immunol. 2025 Nov 25;23(1):31–47. doi: 10.1038/s41423-025-01369-5 (PMC12753682; doi:10.1038/s41423-025-01369-5)

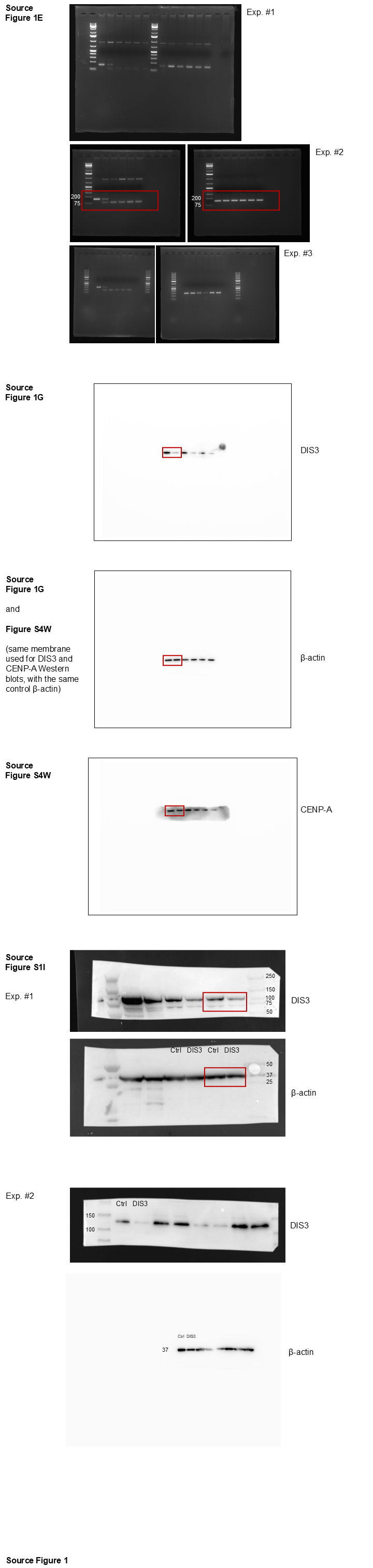

Supplement: Supplementary file 1 — manuscript unmarked [file 41423_2025_1369_MOESM1_ESM.tif]

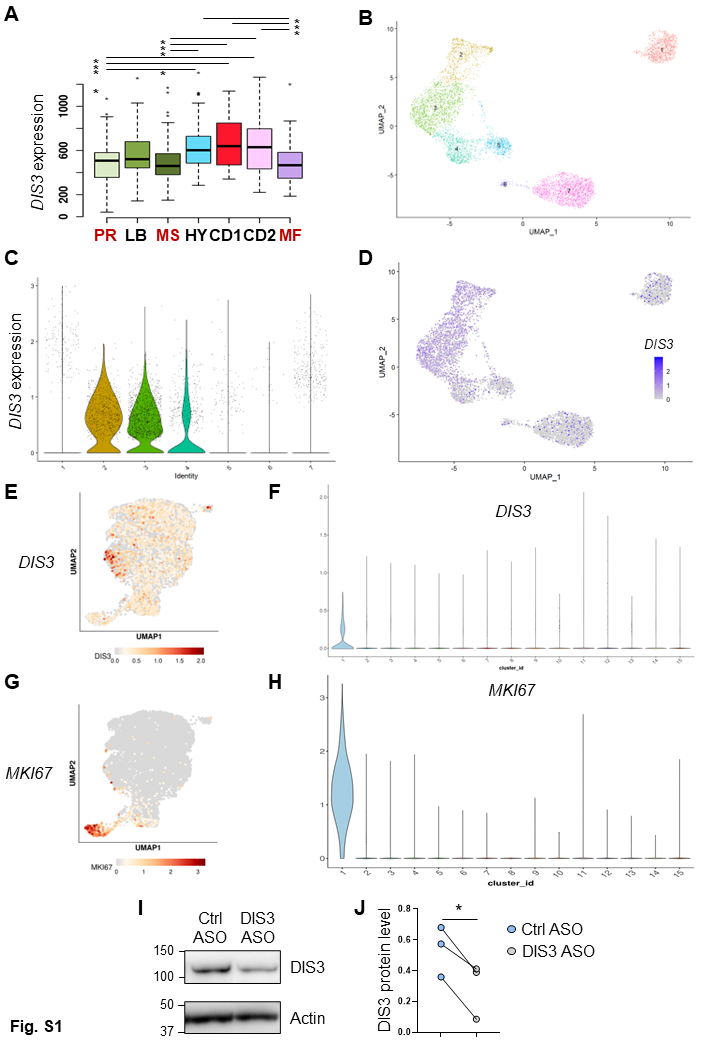

Supplement: Supplementary file 3 — Supplementary Figure S1 [file 41423_2025_1369_MOESM3_ESM.tif]

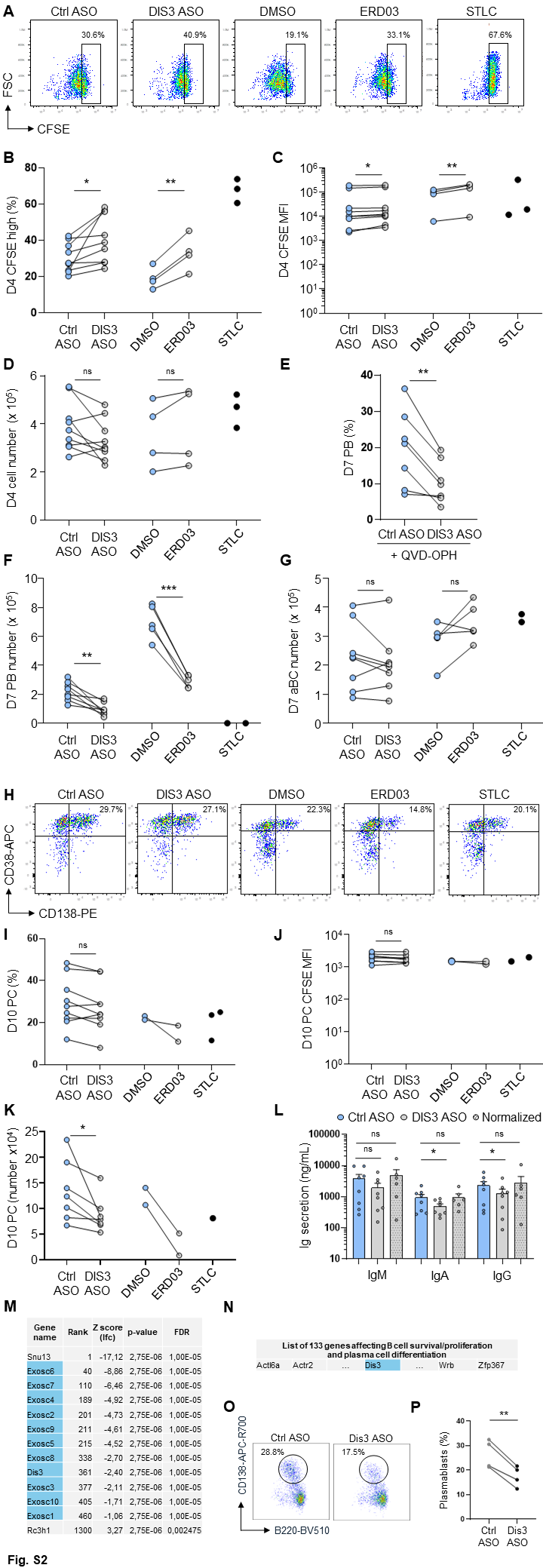

Supplement: Supplementary file 4 — Supplementary Figure S2 [file 41423_2025_1369_MOESM4_ESM.tif]

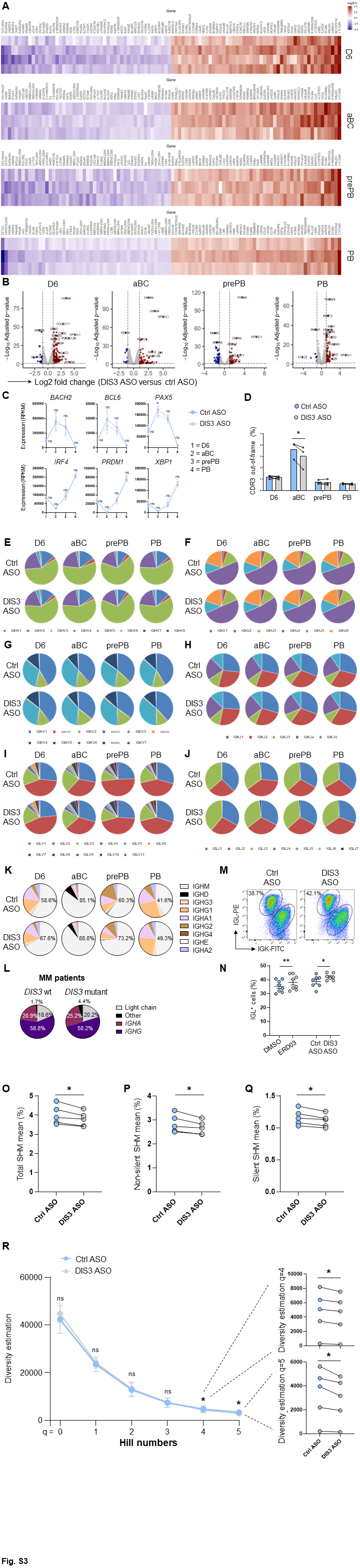

Supplement: Supplementary file 5 — Supplementary Figure S3 [file 41423_2025_1369_MOESM5_ESM.tif]

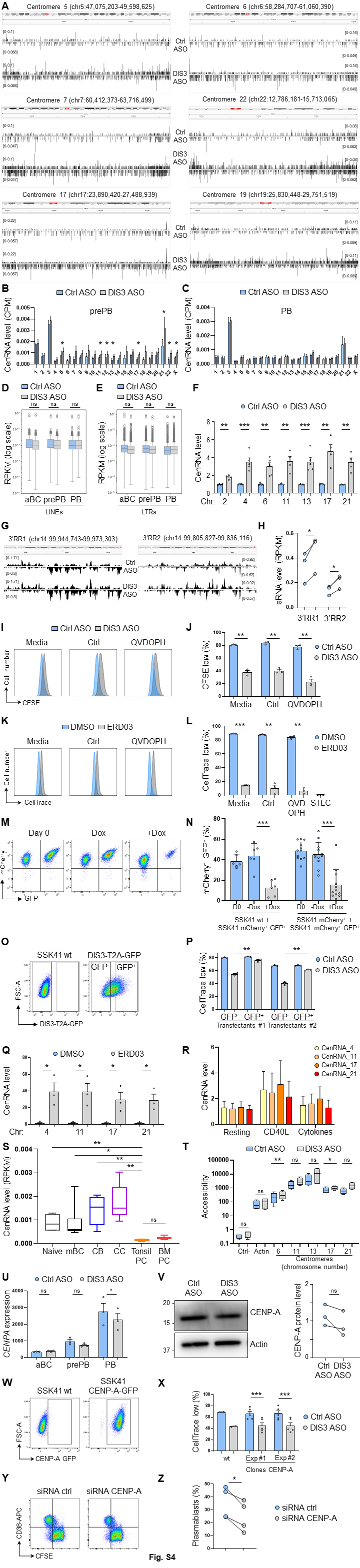

Supplement: Supplementary file 6 — Supplementary Figure S4 [file 41423_2025_1369_MOESM6_ESM.tif]

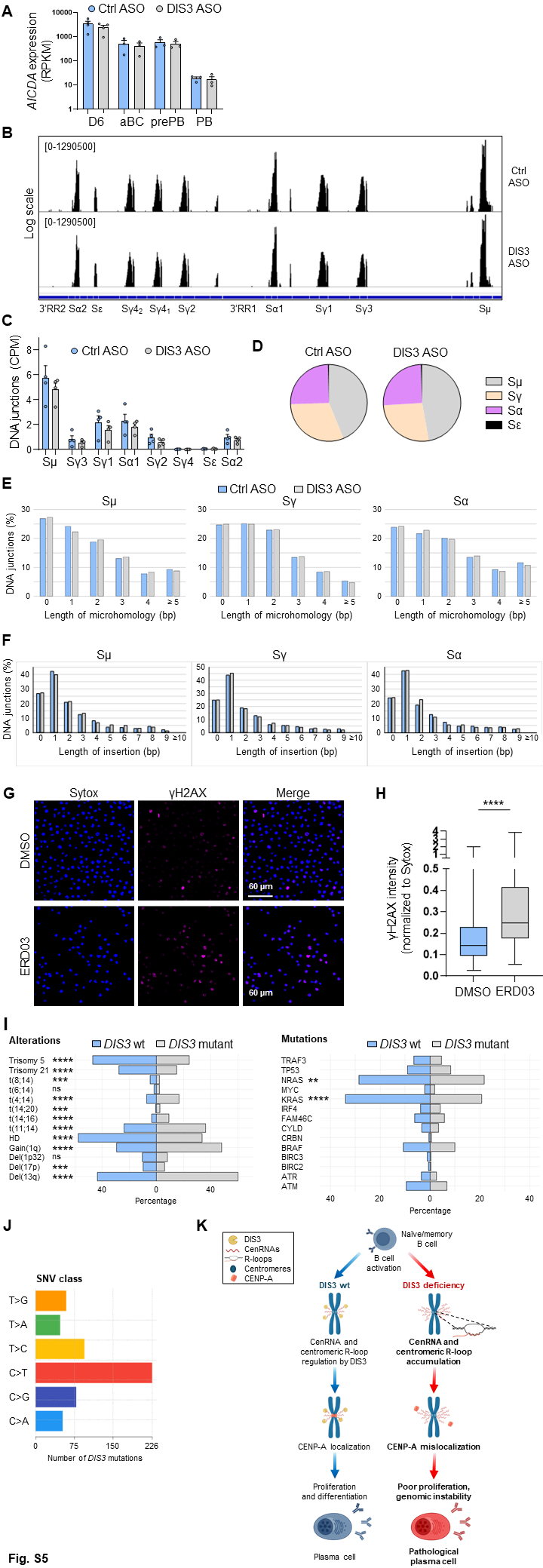

Supplement: Supplementary file 7 — Supplementary Figure S5 [file 41423_2025_1369_MOESM7_ESM.tif]
