# Supplementary material for: DIS3 licenses B cells for plasma cell differentiation in humans
Source: Cell Mol Immunol. 2025 Nov 25;23(1):31–47. doi: 10.1038/s41423-025-01369-5 (PMC12753682; doi:10.1038/s41423-025-01369-5)
Supplement: Supplementary file 2 — Source figure [file 41423_2025_1369_MOESM2_ESM.docx]

## Supplemental Figure Legends

**Figure S1: Transcriptional regulation of *DIS3* in human pathophysiological plasma cells**

**(A)** *DIS3* expression was analyzed in the patients belonging to the 7 groups of the University of Arkansas for Medical Science (UAMS) molecular classification of MM using the TT2 cohort. *DIS3* expression is significantly lower in the PR, MS, and MF groups compared with all the patients of the cohort. Abbreviations: PR, proliferation; LB, low bone disease; MS, t(4;14) translocations; HY, hyperdiploid; CD1, cyclin D1 and cyclin D3 translocations; CD2, cyclin D1 and cyclin D3 translocations; MF, c-MAF and MAFB translocations.

**(B)** UMAP showing single-cell RNA sequencing data from in vitro differentiating cells. 7 clusters were found corresponding to: 1 = memory B cells, 2 and 3 = prePB, 4 and 5 = PB, 6 and 7 = PC.

**(C)** and **(D)** *DIS3* expression in each cluster of single-cell RNA-sequencing from in vitro differentiating cells.

**(E)** UMAP of single-cell RNA-sequencing data from *in vivo* PCs showing *DIS3* expression.

**(F)** *DIS3* expression in each cluster.

**(G)** UMAP of single-cell RNA-sequencing data from *in vivo* PCs showing *MKI67* expression.

**(H)** *MKI67* expression in each cluster.

**(I)** Western blot analyses of DIS3 protein expression after ASO treatments in primary B cells. Cells were treated between D4 and D7 with ctrl or DIS3 ASOs. Data are representative of 3 independent experiments, n=3.

**(J)** Quantification from Western blot analyses of DIS3 protein expression after ASO treatments in primary B cells. Individual values are shown, two-tailed paired *t*-test, 3 independent experiments, n=3.

**Figure S2: DIS3 is mandatory for physiological plasma cell differentiation**

**(A)** DIS3 inhibition decreases B cell proliferation. B cells were treated from D1 to D4 with ctrl or DIS3 ASOs, DMSO or ERD03, or STLC and analyzed by flow cytometry.

**(B)** Quantification of CFSE^high^ cells at D4. Individual values are shown, two-tailed paired *t*-test, at least 4 independent experiments, n=3 to 7.

**(C)** CFSE MFIs were quantified at D4. Individual values are shown, two-tailed paired *t*-test, at least 4 independent experiments, n=3 to 7.

**(D)** Absolute numbers of cells at D4. Individual values are shown, two-tailed paired *t*-test, at least 4 independent experiments, n=3 to 7.

**(E)** Quantification of PBs at D7. Cells were treated with ctrl or DIS3 ASOs in the presence of QVD-OPH. Individual values are shown, two-tailed paired *t*-test, at least 3 independent experiments, n=7.

**(F)** Absolute numbers of PBs at D7. Individual values are shown, two-tailed paired *t*-test, at least 5 independent experiments, n=2 to 8.

**(G)** Absolute numbers of aBCs at D7. Individual values are shown, two-tailed paired *t*-test, at least 5 independent experiments, n=2 to 8.

**(H)** DIS3 inhibition slightly impacts terminal PC differentiation. Cells were treated from D7 to D10 with ctrl or DIS3 ASOs, DMSO or ERD03, or STLC and analyzed by flow cytometry.

**(I)** Quantification of PCs at D10. Individual values are shown, two-tailed paired *t*-test, at least 2 independent experiments, n=2 to 7.

**(J)** CFSE MFIs were quantified at D10 in PCs. Individual values are shown, two-tailed paired *t*-test, at least 2 independent experiments, n=2 to 7.

**(K)** Absolute numbers of cells at D10. Individual values are shown, two-tailed paired *t*-test, at least 2 independent experiments, n=2 to 7.

**(L)** IgM, IgA, and IgG secretion at D10 were evaluated by ELISA. Samples treated with DIS3 ASOs were also normalized to same cell number as the ctrl ASOs (dotted grey bars). Means +/- s.e.m. and individual values are shown, two-tailed paired *t*-test, 8 independent experiments, n=8.

**(M)** All subunits of the RNA exosome complex were found to be essential for mouse PC differentiation.

**(N)** In another independent screening, *Dis3* was found to be one of the 133 genes affecting B cell survival/proliferation and PC differentiation in mice.

**(O)** PC differentiation of mouse primary B cells. Resting B cells were stimulated with LPS for 3 days in the presence of ctrl or Dis3 ASOs before flow cytometry analysis.

**(P)** Quantification of B220^low^ CD138^+^ PBs. Individual values are shown, two-tailed paired *t*-test, 4 independent experiments, n=4.

**Figure S3: Transcriptomic alterations in the absence of DIS3**

**(A)** Heatmaps showing fold changes (FC) of differentially expressed genes from RNA-sequencing at D6, or at D7 in aBCs, prePBs, and PBs. For each condition, the 50 most downregulated and the 50 most upregulated genes, based on the average log2FC with adjusted *P*-values <0.05 across replicates, are shown.

**(B)** Volcano plots of gene expression in the different populations. Red and blue dots correspond to significantly upregulated and downregulated genes, respectively (adjusted *P*-values < 0.05 and |log2FC| > 1).

**(C)** B cell and PC gene expression were analyzed from RNA-sequencing data. Means +/- s.e.m are shown, paired *t*-test, at least 3 independent experiments, n=3 to 4.

**(D)** Out-of-frame CDR3 junctions determined from TRUST4 analyses. Means and individual values are shown, one-tailed paired *t*-test, at least 3 independent experiments, n=3 to 4.

**(E)** to **(K)** TRUST4 repertoire analyses of IGHV **(E)**, IGHJ **(F)**, IGKV **(G)**, IGKJ **(H)**, IGLV **(I)**, IGLJ **(J)**, and IGHC **(K)** gene usage. At least 3 independent experiments, n=3 to 4.

**(L)** Proportions of MM patients with light chain, IGHA^+^, IGHG^+^, or other myeloma in control (*DIS3* wt, n=3999) versus *DIS3* mutant patients (n=545).

**(M)** Flow cytometry analyses of light chain expression. Cells were treated between D4 and D7 with DMSO, ERD03, ctrl or DIS3 ASOs and labelled with anti-κ and anti-λ antibodies at D7.

**(N)** Quantification of λ^+^ cells after ERD03 and ASO treatments. Individual values are shown, two-tailed paired *t*-test, at least 7 independent experiments, n=7 to 8.

**(O)** to **(Q)** DNA repertoire analyses. Total SHM rate was evaluated **(O)**, including non-silent **(P)** and silent mutations **(Q)**. Individual values are shown, two-tailed paired *t*-test, 5 independent experiments, n=5.

**(R)** DNA repertoire analyses. Hill numbers and diversity estimation are shown. **Left:** means +/- s.e.m values are shown. **Right:** individual values are shown. Two-tailed paired *t*-test, 5 independent experiments, n=5.

**Figure S4: DIS3 is necessary for proliferation and centromeric RNAs processing**

**(A)** IGV tracks from RNA-sequencing showing centromeric RNA accumulation at different chromosomes in aBCs treated with ctrl or DIS3 ASO. Data are representative from 3 independent experiments, n=3.

**(B)** Quantification of cenRNA levels at individual chromosomes of prePBs. Means +/- s.e.m. are shown, one-tailed paired *t*-test, 3 independent experiments, n=3.

**(C)** Quantification of cenRNA levels at individual chromosomes of PBs. Means +/- s.e.m. are shown, 3 independent experiments, n = 3.

**(D)** Box plots showing the quantification of LINE transcripts from RNA-sequencing of D6 cells, aBCs, prePBs, and PBs. Two-tailed t-test, 3 independent experiments, n = 3.

**(E)** Box plots showing the quantification of LTR levels from RNA-sequencing of D6 cells, aBCs, prePBs, and PBs. Two-tailed t-test, 3 independent experiments, n = 3.

**(F)** Quantification by RT-qPCR of cenRNA levels after ASO treatments at D6. Means +/- s.e.m. and individual values are shown, two-tailed ratio paired *t*-test, 4 independent experiments, n=4 to 5.

**(G)** IGV tracks from RNA-sequencing showing eRNA accumulation at the 3’RR1 and 3’RR2 in aBCs treated with ctrl or DIS3 ASO. Data are representative of 3 independent experiments, n=3.

**(H)** Quantification of eRNA levels at 3’RR1 and 3’RR2. Individual values are shown, two-tailed paired *t*-test, 3 independent experiments, n=3.

**(I)** SSK41 B cells were incubated in media alone, with DMSO (ctrl), or with QVD-OPH, and treated with ctrl or DIS3 ASOs for 72h. CFSE dye dilution was quantified by flow cytometry.

**(J)** Quantification of cell proliferation after ASO treatments at D3 in SSK41 cells. Means +/- s.e.m. and individual values are shown, two-tailed paired *t*-test, 3 independent experiments, n=3.

**(K)** SSK41 B cells were incubated in media alone, with DMSO (ctrl), or QVD-OPH, and treated with DMSO or ERD03 for 72h. CellTrace dye dilution was quantified by flow cytometry.

**(L)** Quantification of cell proliferation after ERD03 treatments at D3 in SSK41 cells. Means +/- s.e.m. and individual values are shown, two-tailed paired *t*-test, 3 independent experiments, n=3.

**(M)** Competition assays. SSK41 clones expressing Cas9-mCherry alone (control) or Cas9-mCherry + gRNA-GFP (for *DIS3* genetic deletion) were mixed together at day 0. Cells were treated with Dox or not, and analyzed for mCherry and GFP expression by flow cytometry.

**(N)** Quantification of competition assays. Cas9-mCherry gRNA-GFP double positive cells were quantified after competition with SSK41 wt or SSK41 Cas9-mCherry^+^ cells, respectively. 3 clones were analyzed for Cas9-mCherry^+^ cells and 3 clones for Cas9-mCherry^+^ gRNA-GFP^+^ cells. Means +/- s.e.m. and individual values are shown, two-tailed paired *t*-test, 2 (left) and 4 (right) independent experiments, n=3.

**(O)** SSK41 B cells were transfected with a *DIS3-T2A-GFP* expression plasmid. Transfected cells were gated as GFP^-^ and GFP^+^ cells.

**(P)** Quantification of proliferation in GFP^-^ and GFP^+^ cells from the same culture after *DIS3-T2A-GFP* transfection. Means +/- s.e.m. and individual values are shown, two-tailed paired *t*-test, 2 independent experiments with different transfectants, n=3.

**(Q)** Quantification by RT-qPCR of cenRNA accumulation in SSK41 cells after ERD03 treatments. Means +/- s.e.m. and individual values are shown, two-tailed ratio paired *t*-test, 3 independent experiments, n=3.

**(R)** SSK41 B cells were stimulated with CD40L alone or with cytokines before cenRNA quantification by RT-qPCR. Means +/- s.e.m. are shown, 3 independent experiments, n=3.

**(S)** CenRNA expression in human B cells in vivo. RNA-sequencing data were analyzed from naïve B cells (n=4), memory B cells (n=8), centroblasts (CB, n=5), centrocytes (CC, n=7), tonsil PCs (n=4) and bone marrow PCs (n=3). Two-tailed unpaired *t*-test.

**(T)** Chromatin accessibility (ATAC) at centromeres. Primary cells were treated at day 4 with ctrl or DIS3 ASOs and analyzed and day 6. Box and whiskers with minimum to maximum values are values are shown, two-tailed paired *t*-test, 2 independent experiments, n=4.

**(U)** *CENP-A* expression analyzed from RNA-sequencing data of aBCs, prePBs, and PBs. Means +/- s.e.m. and individual values are shown, two-tailed paired *t*-test, 3 independent experiments, n=3.

**(V) Left:** Western blot analyses of CENP-A expression in SSK41 cells after ctrl or DIS3 ASO treatments. Data are representative of 3 independent experiments. **Right:** Quantification of CENP-A protein expression from Western blot analyses. Individual values are shown, two-tailed paired *t*-test, 3 independent experiments, n=3.

**(W)** CENP-A overexpression. SSK41 cells were transfected with a plasmid to express CENP-A-GFP fusion protein, and enriched by cell sorting. Data are representative of 3 independent experiments.

**(X)** Proliferation in CENP-A-overexpressing clones. Proliferation was quantified after 3 days of treatment with ctrl or DIS3 ASOs. Individual values are shown, two-tailed paired *t*-test, 2 independent experiments, n=3.

**(Y)** PC differentiation in CENP-A-deficient cells. Primary B cells were transfected with ctrl or CENP-A siRNAs and plasmablasts were analyzed at day 7.

**(Z)** Quantification of plasmablast differentiation after CENP-A inhibition. Individual values are shown, two-tailed paired *t*-test, 2 independent experiments, n=4.

**Figure S5: Genomic instability in the absence of DIS3**

**(A)** *AICDA* expression analyzed from RNA-sequencing data of D6 cells, aBCs, prePBs, and PBs.

**(B)** Physiological CSR junctions at the *IGH* locus captured by LAM-HTGTS from the Sµ bait. Primary B cells were treated from D4 to D7 with ctrl or DIS3 ASOs before DNA extraction and analyses. Data are representative of 4 independent experiments. Data are represented as log scale.

**(C)** Quantification of physiological CSR junctions at switch regions. Means +/- s.e.m. and individual values are shown, 4 independent experiments, n=4.

**(D)** Proportions of DNA junctions inside the *IGH* locus.

**(E)** DNA junction analysis from LAM-HTGTS experiments. Lengths of micro-homologies in DNA junctions are shown in ctrl and DIS3-deficient cells.

**(F)** DNA junction analysis from LAM-HTGTS experiments. Insertions in DNA junctions are shown in ctrl and DIS3-deficient cells.

**(G)** Microscopy analyses of phospho-γH2AX in DMSO and ERD03 treated cells. SSK41 cells were stained intracellularly with anti-γH2AX antibodies and Sytox, and analyzed by confocal microscopy.

**(H)** Quantification of phospho-γH2AX levels. γH2AX intensity was normalized to Sytox intensity. Box and whisker plots show the median, 25th to 75th percentiles and min to max values, two-tailed unpaired *t*-test, 4 independent experiments, 2761 cells and 2482 cells were analyzed for DMSO and ERD03 treatments, respectively.

**(I)** Genetic alterations in MM patients. Proportions of the genetic alterations in control (*DIS3* wt, n=3077) versus *DIS3* mutant patients (n=520). Two-sided Khi^2^ proportion test. HD = hyperdiploidy.

**(J)** *DIS3* mutations analyses. Transitions and transversions are shown (n=3597).

**(K)** DIS3 is essential for PC differentiation. B cell activation initiates an intense phase of proliferation essential for physiological PC differentiation. We observed that this process was associated with cenRNA expression at centromeric regions. The enzymatic activity of DIS3 was critical for regulating cenRNA levels, ensuring successful cell division and differentiation. In the absence of DIS3 activity, ncRNAs and R-loops accumulate at centromeres, potentially disrupting kinetochore formation by interfering with the recruitment or maintenance of CENP-A, and may alter cell cycle progression. Furthermore, DIS3-deficient cells exhibit decreased DNA recombination but elevated levels of genomic instability and *IGH* translocations, which may contribute to the pathological events leading to PC dysplasia.

a
